# Dosage Effects of Cohesin Regulatory Factor PDS5 on Mammalian Development: Implications for Cohesinopathies

**DOI:** 10.1371/journal.pone.0005232

**Published:** 2009-05-01

**Authors:** Bin Zhang, Jufang Chang, Ming Fu, Jie Huang, Rakesh Kashyap, Ezequiel Salavaggione, Sanjay Jain, Kulkarni Shashikant, Matthew A. Deardorff, Maria L. Giovannucci Uzielli, Dale Dorsett, David C. Beebe, Patrick Y. Jay, Robert O. Heuckeroth, Ian Krantz, Jeffrey Milbrandt

**Affiliations:** 1 Department of Pathology and Immunology, Washington University School of Medicine, St Louis, Missouri, United States of America; 2 Department of Pediatrics, Washington University School of Medicine, St Louis, Missouri, United States of America; 3 Department of Developmental Biology, Washington University School of Medicine, St Louis, Missouri, United States of America; 4 Department of Ophthalmology and Visual Sciences, Washington University School of Medicine, St Louis, Missouri, United States of America; 5 Department of Genetics, Washington University School of Medicine, St Louis, Missouri, United States of America; 6 Department of Medicine (Renal Division), Washington University School of Medicine, St Louis, Missouri, United States of America; 7 HOPE Center for Neurological Disorders, Washington University School of Medicine, St Louis, Missouri, United States of America; 8 Division of Human and Molecular Genetics, The Children's Hospital of Philadelphia, Philadelphia, Pennsylvania, United States of America; 9 Department of Pediatrics, University of Florence, Firenze, Italy; 10 Edward A. Doisy Department of Biochemistry and Molecular Biology, Saint Louis University School of Medicine, Saint Louis, Missouri, United States of America; University Medical Center Groningen, Netherlands

## Abstract

Cornelia de Lange syndrome (CdLS), a disorder caused by mutations in cohesion proteins, is characterized by multisystem developmental abnormalities. PDS5, a cohesion protein, is important for proper chromosome segregation in lower organisms and has two homologues in vertebrates (PDS5A and PDS5B). *Pds5B* mutant mice have developmental abnormalities resembling CdLS; however the role of *Pds5A* in mammals and the association of PDS5 proteins with CdLS are unknown. To delineate genetic interactions between *Pds5A* and *Pds5B* and explore mechanisms underlying phenotypic variability, we generated *Pds5A*-deficient mice. Curiously, these mice exhibit multiple abnormalities that were previously observed in *Pds5B*-deficient mice, including cleft palate, skeletal patterning defects, growth retardation, congenital heart defects and delayed migration of enteric neuron precursors. They also frequently display renal agenesis, an abnormality not observed in *Pds5B^−/−^* mice. While *Pds5A^−/−^* and *Pds5B^−/−^* mice die at birth, embryos harboring 3 mutant *Pds5* alleles die between E11.5 and E12.5 most likely of heart failure, indicating that total *Pds5* gene dosage is critical for normal development. In addition, characterization of these compound homozygous-heterozygous mice revealed a severe abnormality in lens formation that does not occur in either *Pds5A^−/−^* or *Pds5B^−/−^* mice. We further identified a functional missense mutation (R1292Q) in the PDS5B DNA-binding domain in a familial case of CdLS, in which affected individuals also develop megacolon. This study shows that PDS5A and PDS5B functions other than those involving chromosomal dynamics are important for normal development, highlights the sensitivity of key developmental processes on PDS5 signaling, and provides mechanistic insights into how PDS5 mutations may lead to CdLS.

## Introduction

Cornelia de Lange syndrome (CdLS; OMIM #122470) is a rare developmental disorder (∼1∶10,000) characterized by mental retardation, myopia, growth retardation, congenital heart defects, intestinal anomalies, facial dysmorphisms including cleft palate and appendicular skeletal malformations [1]. A breakthrough in the understanding of CdLS occurred when mutations in the *NIPBL* gene were identified in ∼50% of CdLS patients [2], [3]. NIPBL protein is required for cohesin, a protein complex that mediates sister chromatid cohesion, to bind to chromosomes. More recently, mutations in SMC1A and SMC3, core components of cohesin, have been identified in CdLS patients [4], indicating that cohesin dysfunction is the basis of the anomalies associated with CdLS. In addition, mutations in ESCO2, a protein acetyltransferase required for sister chromatid cohesion, have been identified in Robert's syndrome (RBS; OMIM # 268300), a developmental disorder with similarities to CdLS [5], [6]. Most CdLS cases are sporadic (>99%), but some show an autosomal dominant [2], [7] or X-linked recessive pattern of inheritance with variable expressivity or incomplete penetrance [8]. Interestingly, the abnormalities of affected individuals that share the same mutation even within families can vary widely [8]–[11]. The mechanistic basis of variability in phenotypic expressivity and penetrance in CdLS are unknown but are thought to involve modifier genes, epigenetic factors or environmental influences [12].

Cohesin is a highly conserved multi-protein complex required for sister chromatid cohesion, a process that ensures accurate chromosomal segregation during cell division. The cohesin complex consists of the core components, SMC1, SMC3, SCC1(RAD21) and SCC3 (SA), and is regulated by the associated factors, PDS5 (PDS5A and PDS5B in mammals), WAPL, SCC2 (NIPBL), SCC4 (MAU-2), ECO1 (ESCO1 and ESCO2 in mammals) and Separase [13]–[17]. In addition to chromosomal dynamics, cohesin and its regulatory factors play important roles in development by regulating cell movement and axonal outgrowth in *C. elegans* [18], [19], transcriptional regulation of neurodevelopmental regulators in *Zebrafish* [20], and control of long-range gene expression in *Drosophila* [21]. Most recently, the non-mitotic functions of cohesin and cohesin-associated proteins have been highlighted by their high expression in post-mitotic neurons in adult mice [22], [23], the neuronal deficits in *Pds5B*-deficient mice [22], and the discovery that *Drosophila* mutants in *SMC1*, *RAD21*, *and SA* have defects in axonal pruning and dendritic targeting [24], [25]. These studies, coupled with the identification of causative mutations in cohesin components in human developmental syndromes, promise to shed new light on the non-canonical functions of cohesin and cohesin-associated proteins during development.

While early work in *Drosophila* indicated that Nipped-B (NIPBL, SCC2) is important for regulating long-range gene expression of the *cut* locus [26], the role of cohesin in transcription has been difficult to decipher. A recent genome-wide study in *Drosophila* showed that Nipped-B and cohesin bind to the transcribed and regulatory regions of many active developmental genes [27], providing further evidence for cohesin-mediated transcriptional regulation. ChIP-on-Chip analysis of cohesin binding sites across the human genome and 3% of the mouse genome revealed that cohesin is bound to chromatin at ∼9,000 sites and, that most of these sites overlap with binding sites for the chromatin insulator protein CTCF [23], [28]. The co-localization of cohesin and CTCF suggests that a possible molecular mechanism underlying developmental disorders caused by cohesin component mutations is the aberrant regulation of gene expression. The molecular features of CTCF (a transcriptional insulator) and cohesin, which topologically encircles chromosomes [29], suggest that together they may regulate gene expression through control of global nuclear dynamics, such as interchromosomal or intrachromosomal looping. The contribution of cohesin to CTCF-mediated insulator activity [23] could account for the variability in phenotypic expressivity and penetrance observed in CdLS.

The molecular functions of PDS5A and PDS5B in mammals are largely unknown, but their homologues in lower organisms are required for sister chromatid cohesion and interact genetically and/or physically with cohesin core components and its accessory factors [30]. In fungi, PDS5 antagonizes the establishment of cohesion until counteracted by ECO1 [31] and is acetylated by ECO1 *in vitro* [32]. PDS5A and PDS5B are very similar except for the addition of two AT-hook motifs at the carboxy terminus of PDS5B. PDS5A and PDS5B both interact with WAPL, a cohesin-associated protein that regulates dissociation of cohesin from chromatin [16].

Previously, we generated and characterized *Pds5B*-deficient mice and found that they die at birth with a constellation of abnormalities reminiscent of those observed in patients with CdLS [22]. The high frequency of mental retardation and behavioral disorders in CdLS suggests that cohesin abnormalities lead to neuronal dysfunction. In accord, we found high expression of PDS5B in post-mitotic neurons as well as deficits in neuronal migration and axonal projection in *Pds5B*-deficient mice. We also noted the absence of sister chromatid cohesion deficits in cells from these mice, again consistent with findings in CdLS patients. Overall, these observations indicate that cohesin proteins have multiple roles that together shape the development of multi-cellular organisms.

Similar to human CdLS, *Pds5B* mutant mice exhibit variable expressivity of phenotypes, but unlike human CdLS, *Pds5B* mutant mice do not have diaphragmatic hernia or eye or kidney abnormalities, which are associated with CdLS. It is possible that *Pds5A* may have compensated for *Pds5B* loss or these defects may be dependent solely on *Pds5A* activity or require marked reduction in total PDS5 activity. To explain the variable expressivity observed in *Pds5B*-deficient mice and CdLS cases, we hypothesized that *Pds5A* may be partially compensating for *Pds5B* loss. To this end, we generated and characterized *Pds5A*-deficient mice and found that they manifest multiple developmental defects. Many of the abnormalities are similar to those present in CdLS patients and *Pds5B*-deficient mice, suggesting functional redundancy between PDS5A and PDS5B in embryonic development. The CdLS-like developmental anomalies in *Pds5A*, *B*-deficient mice along with the involvement of PDS5A and PDS5B in the same molecular complex as NIPBL, SMC1A, SMC3, prompted us to explore whether *PDS5A* and *PDS5B* mutations are associated with CdLS. We sequenced the *PDS5A* and *PDS5B* gene from genomic DNAs of *NIPBL* and *SMC1A* mutation negative CdLS patients and identified a missense mutation (R1292Q) that disrupts PDS5B DNA-binding and likely causes or contributes to a familial case of CdLS. These findings provide strong evidence that the functions of PDS5 proteins and associated cohesin are crucial for normal mammalian development.

## Results

### *Pds5A* deficiency results in developmental abnormalities similar to those present in *Pds5B* mutant mice

Because *Pds5B* mutant mice exhibit phenotypic variability and incomplete penetrance [22], we hypothesized that *Pds5A* might partially compensate for mutant *Pds5B* function. To test this hypothesis and to determine the role of *Pds5A*, we generated *Pds5A*-deficient mice using ES cells with an insertion of the β-geo transgene in the second intron (Baygenomics genetrap line RRM243, See Fig. S1 in Supplementary Data and Materials S1). Quantitative RT-PCR analysis revealed universal expression of both *Pds5A* and *Pds5B* during embryonic development (See Fig. S2 in Supplementary Data and Materials S1). In adulthood, *Pds5A* is expressed at similarly low levels in all tissues (See Fig. S3A in Supplementary Data and Materials S1), whereas adult expression of *Pds5B* is more variable between tissues, with particularly high levels observed in adult testis and brain [22]. Using the *Pds5A*-deficient mice, we also examined the *Pds5A* expression pattern using X-gal histochemistry to detect the mutant *Pds5A*-β-galactosidase fusion protein created by gene trap. We detected expression of β-galactosidase in most tissues, including the nervous system where Purkinje cells in the cerebellum and neurons in the cerebral cortex and retina had strong X-gal staining (See Fig. S3 in Supplementary Data and Materials S1). We also found high *Pds5A* mRNA and protein levels in cultured dorsal root ganglia and hippocampal neurons, supporting the idea that PDS5 proteins play important roles in post-mitotic neurons (data not shown).

**Figure 1:**
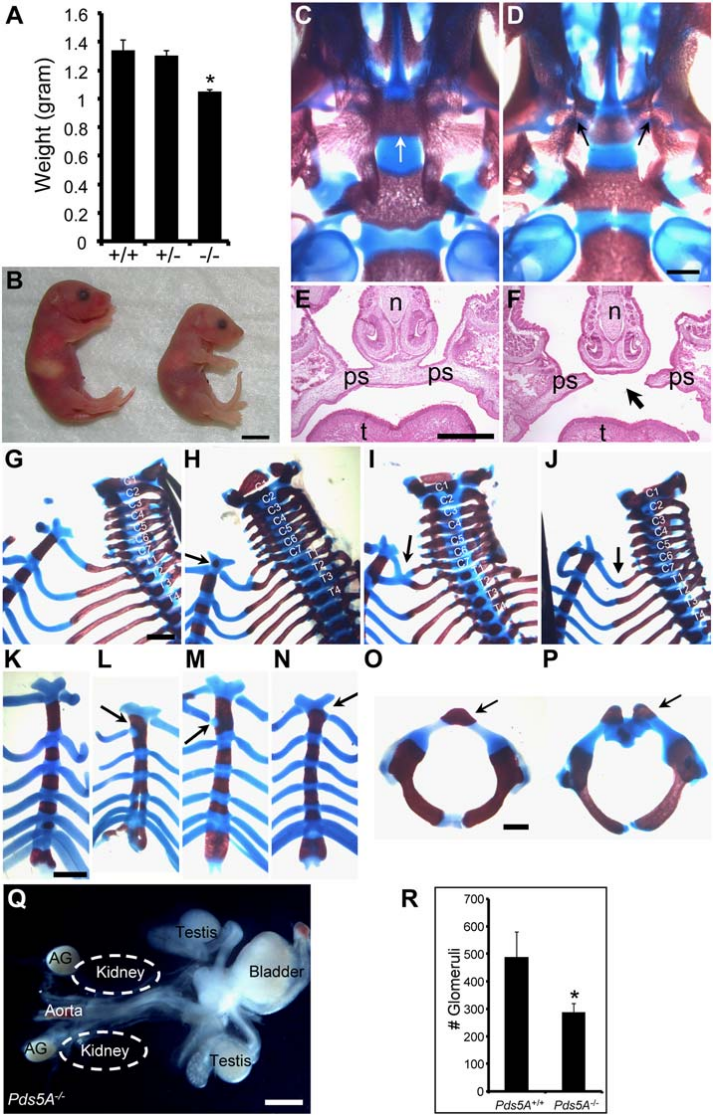
*Pds5A* deficiency results in growth retardation, abnormal skeletal patterning and cleft palate. (A) The weight of *Pds5A^−/−^* P0 mice is significantly lower than that of wildtype and heterozygous littermates (WT, *n* = 8; *Pds5A^+/−^*, *n* = 30; *Pds5A^−/−^*, *n* = 39). Error bars represent s.e.m. ^*^ *P*<0.001, Student's unpaired, two tailed *t*-test. (B) Morphology of wildtype and *Pds5A* mutant mice. Note that newborn *Pds5A^−/−^* pups (right) were smaller than their wildtype littermates (left). Note too the absence of milk in the stomach of the *Pds5A^−/−^* pup. Scale bar: 0.5 cm. (C, D) Alizarin Red S and Alcian Blue staining of neonatal skulls demonstrates complete cleft palate (arrows) of a *Pds5A^−/−^* neonate (D) compared with the proximity of palatal bones in the midline (arrow) in the wildtype control (C). Scale bar: 0.5 mm. (E, F) Palatogenesis defects illustrated using H&E stained coronal sections of E18.5 wildtype (E) and *Pds5A^−/−^* (F) embryos. ps, palatal shelf; n, nasal bone; t, tongue. Arrows in F point to the unfused palatal shelves. Scale bar: 1 mm. (G–P) Alizarin Red S and Alcian Blue staining of newborn skeleton. Bone is stained red and cartilage is stained blue. (G–J) Cervical and thoracic vertebrae and ribs. Note the C7-T1 fusions (arrows in H, I) and cervical rib (arrow in J) in *Pds5A^−/−^* mice, compared to the normal skeletal patterning in wildtype animals (G). Cervical (C) and Thoracic (T) vertebrae are marked by numbers. (K–N) Sternum morphology. Arrows denote ectopic rib-sternum conjunctions (L–N) in *Pds5A^−/−^* mice compared to sternum patterning in wildtype (K). (O, P) Morphology of the first cervical vertebrae. Note the unfused ossification centers at the dorsal tip of the first cervical vertebrae (arrows) in *Pds5A^−/−^* animal (P) compared to wildtype (O). Scale bar for G–N: 1 mm; scale bar for O–P: 0.5 mm. (Q, R) Renal abnormalities in neonatal *Pds5A*-deficient mice. (Q) Bilateral renal agenesis in *Pds5A^−/−^* newborn animals. AG, adrenal gland. Scale bar: 1 mm. (R) The total number of glomeruli in kidneys from P0 *Pds5A^−/−^* and wildtype mice were counted (*n* = 4 animals, 8 kidneys). (^*^, *P*<0.05, student unpaired *t* test; mean±s.e.m.).

To determine the impact of *Pds5A* loss during development we bred heterozygous *Pds5A* animals to obtain *Pds5A* null animals. We found that *Pds5A*-deficient mice die perinatally, and are significantly smaller than their wildtype and *Pds5A*-heterozygous littermates (Fig. 1A), even though no overt structural abnormalities were observed (Fig. 1B). To examine the skeleton, we stained newborn pups with Alcian Blue and Alizarin Red S to highlight the cartilage and bones. We detected several defects with incomplete penetrance and variable expressivity including cleft palate (20/61 in *Pds5A^−/−^*, 4/77 in *Pds5A^+/−^*, 0/39 in *Pds5A^+/+^*; Fig. 1C–F), presence of cervical ribs (10/10 in *Pds5A^−/−^*, 0/11 in *Pds5A^+/+^*; fusion of C7 and T1 or transformation of C7 to T1; Fig. 1G–J), abnormal sternum (5/10 in *Pds5A^−/−^*, 0/25 in *Pds5A^+/+^*; Fig. 1K–N) and abnormal patterns of vertebral ossification (17/26 in *Pds5A^−/−^*, 0/7 in *Pds5A^+/−^*, 0/3 in *Pds5A^+/+^*; Fig. 1O, P). Many of these defects were similar to those observed in *Pds5B* mutant mice, indicating that both PDS5 proteins have important roles in skeletal patterning and palatogenesis. Interestingly, both mouse mutants had variable penetrance and expressivity of these skeletal defects suggesting that total *Pds5* dosage is critical for palatogenesis and skeletal patterning. The importance of *Pds5* dosage is further highlighted by the frequency of cleft palate, which is 33% (20/61) in *Pds5A* homozygotes and 5% (4/77) in *Pds5A* heterozygotes, a finding consistent with the idea that reduced dosage of functional cohesin results in the developmental anomalies characteristic of CdLS.

Renal anomalies are also associated with CdLS [1] and PDS5A has been implicated in renal growth [33]. These reports and the high renal expression of *Pds5A* in newborn and adult mice (See Fig. S3A, F in Supplementary Data and Materials S1) prompted us to examine renal development in *Pds5A*-deficient mice. We found that ∼13% (8/61) of *Pds5A*-deficient mice have either unilateral or bilateral kidney agenesis (Fig. 1Q). Furthermore, when kidneys do develop in these mutant mice, they are hypoplastic and have a reduced number of glomeruli (Fig. 1R). The defects in embryonic renal development could contribute to the perinatal lethality of the *Pds5A*-deficient animals.

### *Pds5A*-mutant mice have normal sympathetic neuronal projections and germ cell differentiation

**Figure 2:**
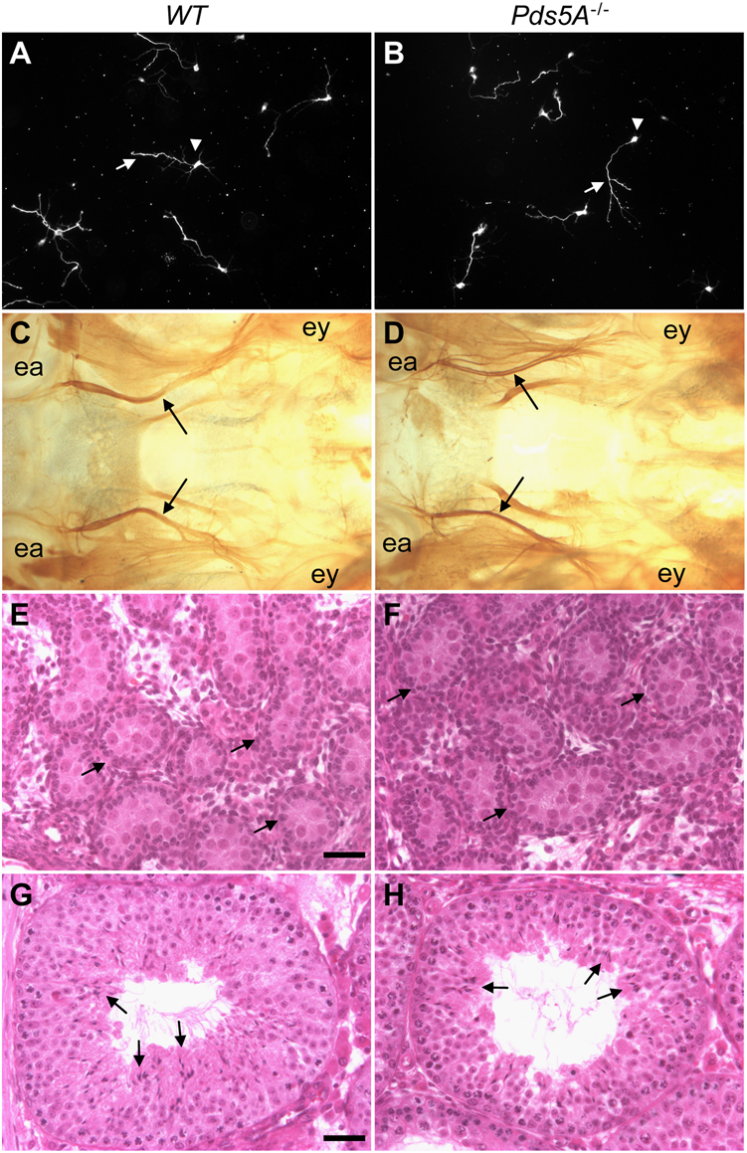
Normal development of hippocampal neurons, the superior cervical ganglion, and germ cells in *Pds5A* ^−/−^ mice. (A, B) Hippocampal neurons from wildtype and *Pds5A* mutant mice were cultured for 3 days and stained with TuJ1 antibody. The neurons show normal axonal projection and polarity (one axon per neuron). Arrowheads, cell bodies; arrows, axons. (C, D) Tyrosine hydroxylase (TOH) whole-mount staining of sympathetic projections from the superior cervical ganglion in wildtype (C) and *Pds5A* mutant (D) mice showed that the carotid nerves (indicated by arrows) project normally to the eye. ey, eye; ea, ear. (E, F) H&E staining of germ cells in neonatal testes showed similar numbers of germ cells in wildtype (E) and *Pds5A* ^−/−^ (F) mice. Arrows point to testicular cords surrounding the germ cells. (G, H) H&E staining of 6-week testes transplants of E18.5 wildtype (G) and *Pds5A^−/−^* mice (H). Note the presence of elongated spermatids with condensed heads and long tails in both wildtype and *Pds5A^−/−^* testis explants (arrows).

Cohesin components are crucial for axonal projection and neuronal migration in mice (PDS5B) [22], axonal pruning and dendritic targeting in *Drosophila* (SMC1, RAD21, and SA) [24], [25], axonal guidance in *C. elegans* (MAU-2/SCC4) [18], and nervous system development in zebrafish (SMC1, RAD21) [20]. The expression of *Pds5A* in post-mitotic neurons (See Fig. S3 in Supplementary Data and Materials S1), the role of other cohesin components in neuronal development and function, and the mental retardation and behavioral abnormalities associated with CdLS, encouraged us to examine the nervous system in *Pds5A* ^−/−^ mice. Consistent with the largely normal brain anatomy observed in CdLS patients and in *Pds5B*-deficient mice, we did not observe any gross or microscopic structural anomalies in *Pds5A* ^−/−^ brain. Cultured hippocampal neuron developed normally (data not shown) with no obvious defects in survival or polarity (Fig. 2A and B). These data suggest that PDS5A does not play obvious roles in embryonic central nervous system development.

The defects in sympathetic innervation emanating from the superior cervical ganglion (SCG) observed in *Pds5B*-deficient mice could explain the ptosis frequently associated with CdLS [1]. In contrast to *Pds5B*-deficient mice, the sympathetic nervous system in *Pds5A^−/−^* mice was unremarkable, with a well developed SCG that was positioned appropriately and had normally appearing neuronal projections (Fig. 2C and D).

*Pds5B*-deficient mice have severe depletion of primordial germ cells in the testes and ovaries and some cases of CdLS also exhibit significant reduction or absence of germ cells [22], [34]. An examination of *Pds5A^−/−^* mice revealed normal germ cell development with similar numbers of germ cells in both the testes and ovaries of wildtype and *Pds5A^−/−^* P0 mice (Fig. 2E and F; See Fig. S4 in Supplementary Data and Materials S1). To further examine whether PDS5A might regulate sister chromatid cohesion during meiosis, a histological analysis of gonadal germ cells in P0 female mice was performed. We found that most germ cells in both *Pds5A*-deficient and wildtype ovaries demonstrated the distinctive nuclear morphology with condensed chromosomes that characterize the zygotene and pachytene stages of meiotic prophase (See Fig. S4A, B in Supplementary Data and Materials S1). We also examined spermatogenesis, using testicular transplantation [22] to propagate E18.5 testis as explants on the backs of nude mice. We found similar numbers of spermatogonia and round spermatids in wildtype and *Pds5A* mutant transplanted testes 4 weeks after transplantation (n = 4; See Fig. S4C,D in Supplementary Data and Materials S1). By 6 weeks after transplantation, the explanted *Pds5A^−/−^* testes contained testicular cords with the full spectrum of spermatogenesis (n = 5; Fig. 2 G, H), including mature spermatids with condensed heads and elongated tails (See Fig. S4E,F in Supplementary Data and Materials S1). Together, these data indicated that *Pds5A* is not required for meiosis or primordial germ cell development.

### *Pds5* gene dosage affects mouse embryonic development: phenotypic defects in *Pds5A* ^+/−^; *Pds5B^−/−^* mice and *Pds5A^−/−^*;*Pds5B^+/−^* mice

**Figure 3:**
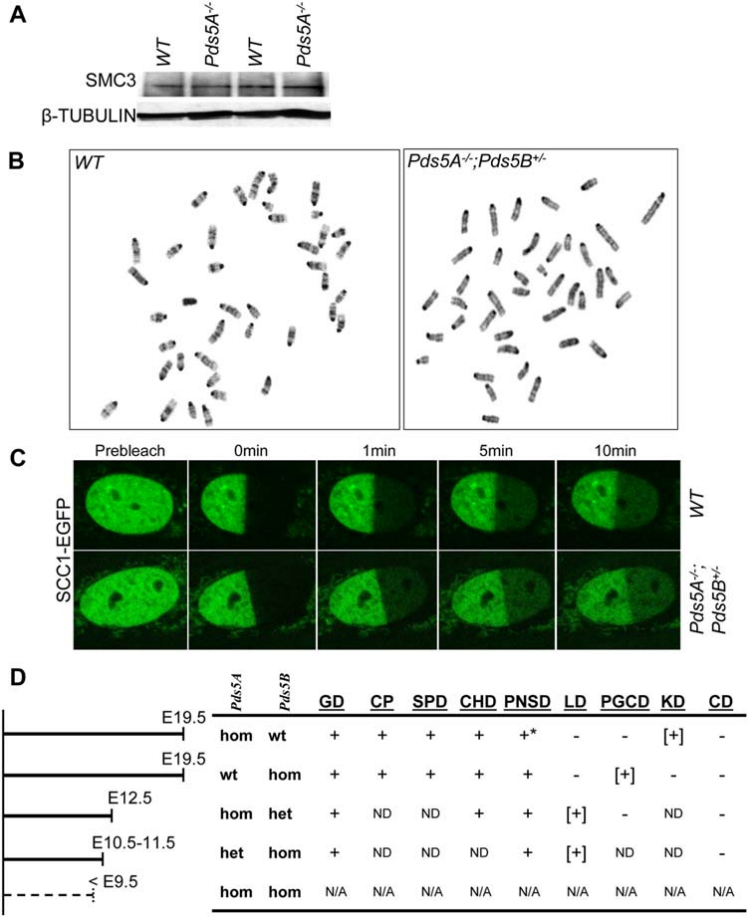
*Pds5* gene dosage synergistically affects development but does not cause defects in sister chromatid cohesion or cohesin-chromatin association. (A) Overall cohesin levels in E14.5 kidney lysates were assessed by Western blotting using anti-SMC3 antibodies. ß-tubulin levels were used to confirm equivalent protein loading. Note that SMC3 levels were equivalent in tissues from wildtype and *Pds5A^−/−^* mice. (B) GTG (giemsa-banding using trypsin and giemsa) banded metaphase spreads from mouse embryonic fibroblasts (MEFs) derived from wildtype and *Pds5A^−/−^*;*Pds5B^+/−^* mice showed no evidence of precocious sister chromatid separation (PSCS). (C) Fluorescent Recovery After Photobleaching (FRAP) using SCC1-EGFP in wildtype and *Pds5A^−/−^*;*Pds5B^+/−^* MEFs. Half of the nucleus was bleached and fluorescence intensity was measured every 30 sec for 30 min. The images show the recovery dynamics over time. Note no significant difference of cohesin recovery dynamics between wildtype and *Pds5A^−/−^*;*Pds5B^+/−^* cells after photobleaching. (D) A summary table listing the abnormalities associated with *Pds5* deficiencies demonstrates the genetic interactions between *Pds5A* and *Pds5B*. The left diagram displays the age when most embryos of the indicated genotype died. The right table lists the phenotypes observed in the indicated embryos. Hom, homozygote; het, heterozygote; wt, wild type; GD, growth delay; CP, cleft palate; SPD, skeletal patterning defects; CHD, congenital heart defects; PNSD, peripheral nervous system defects; LD, lens development defects; PGCD, primordial germ cell defects; CD, cohesion defects; KD, kidney developmental defects. +, presence of the indicated phenotype; - absence of the indicated phenotype; ND, not determined; N/A, not applicable since no embryos were obtained for double homozygous mutants. *, *Pds5A* homozygotes have delayed ENS precursor migration, but normal SCG projection. [+], highlights functional redundancy and diversification of PDS5A and PDS5B.

*Pds5B*-deficient cells do not display defects in chromosome cohesion, suggesting that the developmental abnormalities in *Pds5B* mutant mice arise from the lack of other, non-canonical PDS5B functions [22]. To test for cohesion defects in *Pds5A*-deficient cells, we obtained MEF lines (n = 3) from *Pds5A*-deficient and wildtype embryos. Both *Pds5A*-deficient and wildtype MEFs proliferate at the same rate (data not shown), and the levels of the cohesin components SMC3 (Fig. 3A) and SCC1 in these cells were equivalent (data not shown). We performed metaphase chromosome analysis by GTG (giemsa-banding using trypsin and giemsa) banding of these mutant MEFs. We analyzed >50 metaphase cells per line and found no obvious cohesion defects such as precocious sister chromatid separation (PSCS) or gross chromosomal abnormalities in *Pds5A^−/−^* MEFs (data not shown). The lack of sister chromatid cohesion abnormalities in cells lacking either PDS5A or PDS5B suggests that they have redundant functions in sister chromatid cohesion and that their roles in mammalian embryonic development are unlikely to be related to their role in cohesion.

The phenotypic overlap between *Pds5A-* and *Pds5B*-deficient mice also suggests the possibility of redundant functions between these two closely related homologs during development. To study the effects of PDS5 dosage on sister chromatid cohesion and embryonic development, we crossed *Pds5A^+/−^* with *Pds5B^+/−^* to obtain compound heterozygotes. These were then used to generate double homozygous or compound homozygous-heterozygous mice (i.e. mice with only one wild type *Pds5* allele, either *Pds5A* or *Pds5B*). No *Pds5A^−/−^*;*Pds5B^−/−^* double homozygous embryos were obtained at E9.5 (Fig. 3D), indicating that depletion of both *Pds5A* and *Pds5B* leads to very early embryonic lethality. However, *Pds5A^+/−^*;*Pds5B^−/−^* and *Pds5A^−/−^*;*Pds5B^+/−^* embryos were identified, but had severe growth retardation and died between E11.5 and E12.5. The phenotype of these mutant embryos clearly demonstrated the redundant effects of PDS5A and PDS5B on heart, enteric nervous system, and lens development (Fig. 3D).

To ascertain whether low levels of PDS5 proteins can affect sister chromatid cohesion, cohesin stability or cohesin chromosome-binding dynamics, we studied MEFs prepared from *Pds5A^−/−^*;*Pds5B^+/−^* and *Pds5A^+/−^*;*Pds5B^−/−^* embryos. The analysis of metaphase spreads of these MEFs did not show any abnormalities in sister chromatid cohesion (Fig. 3B). We then used fluorescence recovery after photobleaching (FRAP) assays to compare the dynamics of association of a SCC1-EGFP fusion protein with chromosomes in mutant and wildtype MEFs. We found that fluorescence recovery dynamics of SCC1-EGFP in wildtype, *Pds5A^−/−^;Pds5B^+/−^*, and *Pds5A^+/−^;Pds5B^−/−^* MEFs all showed similar slow recovery curves (Fig. 3C; See Fig. S5A in Supplementary Data and Materials S1) with a time scale consistent with previous reports [35]. We also found no significant differences in residence times of either weak or strong chromatin-bound SCC1-EGFP between wildtype and *Pds5* mutant MEFs (See Fig. S5B in Supplementary Data and Materials S1). There is a similar composition of soluble SCC1-EGFP as well as weak and strong chromatin-bound SCC1-EGFP proteins between wildtype and *Pds5* mutant MEFs (See Fig. S5C in Supplementary Data and Materials S1) was also observed. These results indicate that cells with only one *Pds5* wildtype allele (either *Pds5A* or *Pds5B*) have normal cohesin stability and cohesin-chromatin association dynamics and imply that embryonic development is more sensitive than sister chromatid cohesion to levels of PDS5 activity.

### Both PDS5A and PDS5B are important for cardiac development

**Figure 4:**
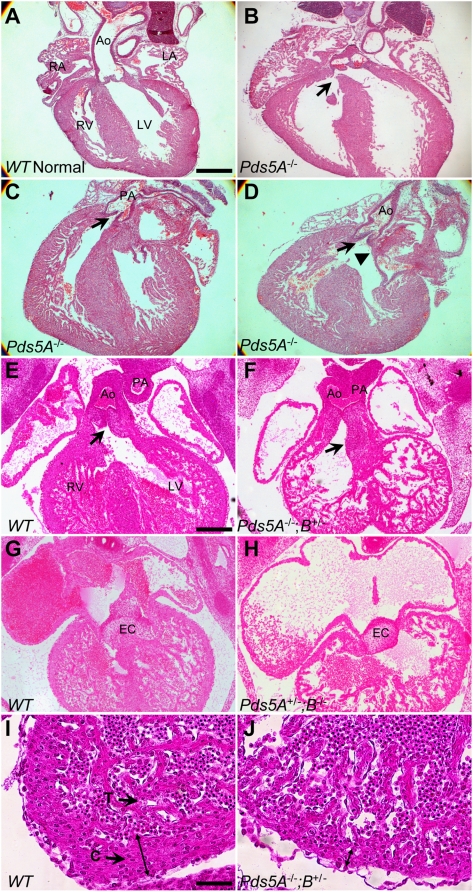
*Pds5A* mutant mice manifest cardiac abnormalities similar to those observed in CdLS. (A) A section of a wildtype mouse heart demonstrates the intact ventricular septum and its relationship to the aortic and tricuspid valves.. (B–D) Sections of *Pds5A* mutant hearts showing: (B) a perimembranous ventricular septal defect (VSD); (C) the pulmonary valve and artery in a heart with double outlet right ventricle; (D) the aorta arising from the right ventricle of the same heart with a muscular conus between the aortic and mitral valve (arrowhead). Arrows indicate the respective defects. Scale bar: 0.5 mm. (E–J) Severe cardiac defects in *Pds5A^+/−^;Pds5B^−/−^* and *Pds5A^−/−^;Pds5B^+/−^* embryos. (E) A section from wildtype E12.5 embryonic heart shows the separation of aorta and pulmonary artery and the developing aortic valves (arrow). (F) A *Pds5A^−/−^;Pds5B^+/−^* E12.5 embryonic heart shows the underdeveloped endocardial cushion of the outflow tract and the non-septated aorta and pulmonary artery (i.e., truncus arteriosus). (G) Normal association of the atrial and ventricular septae with the endocardiac cushion and valve morphogenesis in a wildtype E12.5 heart. (H) A *Pds5A^+/−^;Pds5B^−/−^* E12.5 heart demonstrates the dilated atria and underdeveloped endocardial cushion with no valve formation, and failure of atrioventricular canal septation. (I) Wildtype E12.5 heart with a compact myocardial layer of normal thickness. (J) A *Pds5A^−/−^;Pds5B^+/−^* heart has a thin compact myocardium. Double-headed arrows in (I, J) indicate the compact myocardial layer. Ao, aorta; PA, pulmonary artery; RA, right atrium; LA, left atrium; RV, right ventricle; LV, left ventricle; EC, endocardial cushion; T, trabecular myocardium; C, compact myocardium.

Congenital heart defects (CDH), primarily atrial septal defects, are frequently observed in children with CdLS (range 14–70% in different studies) [36]. Similar types of CHDs are present in *Pds5B^−/−^* mice [22]. *Pds5A^−/−^* mice suffer from respiratory distress and die within a few hours after birth, suggesting that they may also have cardiac anomalies. Indeed, we found that *Pds5A^−/−^* mice, compared to wildtype mice, had significantly higher incidence of congenital heart defects (17/25 versus 3/18, P<0.001; Fig. 4A–D, See Table S1 in Supplementary Data and Materials S1), including those that are commonly found in CdLS patients [1] and *Pds5B*-deficient mice [22].

In *Pds5A^−/−^* mice, we identified atrial septal defect of the secundum type (ASD, 1/25), isolated ventricular septal defects (VSDs, 10/25; all perimembranous), double outlet right ventricle (DORV, 1/25), and atrioventricular canal defects (AVCDs, 5/25) (Fig. 4A–D; See Table S1 in Supplementary Data and Materials S1). All of these defects, except for ASD, would be expected to cause excessive pulmonary circulation and edema from left-to-right shunting of blood at the ventricular level, thus resulting in respiratory distress and potentially contributing to the early postnatal lethality.

Heterozygous *Pds5A* mice (5/19) also had heart defects that were more severe than those in wildtype littermates. For instance, we identified *Pds5A* heterozygotes with tetralogy of Fallot (TOF, a common malformation in CdLS, which was also associated with an ASD; 1/19), ASD alone (3/19), or VSD (1/19) (See Table S1 in Supplementary Data and Materials S1).

Interestingly, we identified small VSDs in 3/18 wildtype littermates derived from *Pds5A* heterozygote breedings. This is significantly higher than seen in wild-type controls in other mouse models of congenital heart disease (<1%; Jay PY, unpublished observations). This observation suggests that parental *Pds5A* heterozygosity could adversely affect the genetic stability of gametes, perhaps by causing aneuploidy or epigenetic changes that increase the frequency of developmental defects in the progeny. This hypothesis is supported by the recent evidence that maternal CTCF, whose function may be mediated via cohesin complexes [23], [28], is essential for proper oocyte meiosis and embryonic development [37].

The high expression of both *Pds5A* and *Pds5B* in the embryonic heart along with the presence of cardiac defects in both *Pds5A*- and *Pds5B*-deficient mice emphasizes the crucial function of PDS5 activity in cardiac development. To further explore the consequences of PDS5 loss in the heart, we examined *Pds5A^−/−^;Pds5B^+/−^* or *Pds5A^+/−^;Pds5B^−/−^* E12.5 embryos. All embryos lacking three *Pds5* alleles (n = 4) had severe cardiac abnormalities including truncus arteriosus (TA) and a common atrioventricular canal. The endocardial cushions of the outflow tract failed to develop separate aortic and pulmonic valve leaflets, and the aorticopulmonary septum was absent (Fig. 4E, F). An underlying defect in neural crest-derived cell migration likely contributes to these abnormalities of outflow tract development [38]. The common atrioventricular canal defect was marked by severe hypoplasia or aplasia of the ventricular septum and a failure of valve development from the atrioventricular canal cushion tissue (Fig. 4G, H). Furthermore, all *Pds5A^−/−^;Pds5B^+/−^* or *Pds5A^+/−^;Pds5B^−/−^* embryos had a thin compact ventricular myocardium and dilated atria (Fig. 4I, J). The absence of competent atrioventricular valves likely causes atrial dilation due to regurgitant blood flow. In combination with a thin, dysfunctional myocardium, forward cardiac output would be severely compromised, leading to growth retardation and embryonic lethality.

### Synergistic effects of PDS5A and PDS5B on enteric nervous system (ENS) development

**Figure 5:**
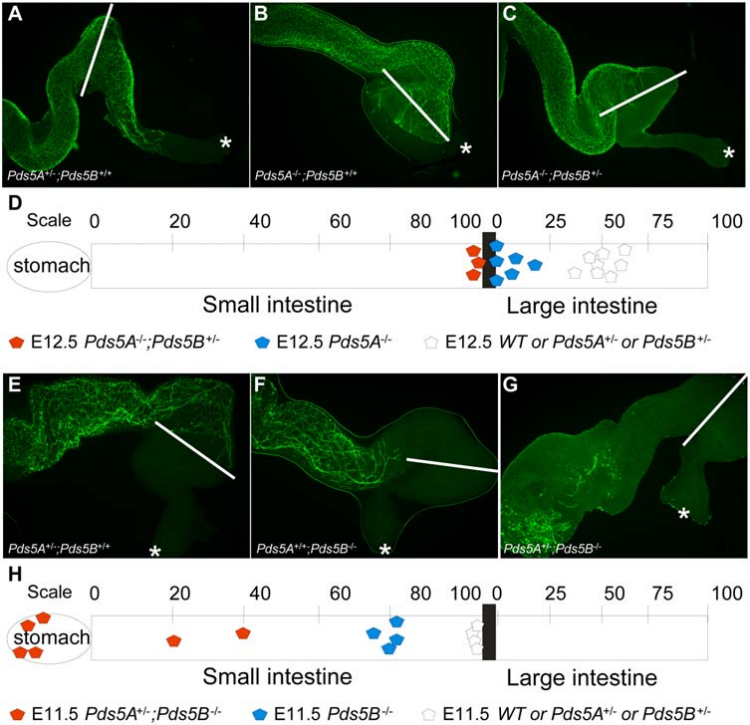
Synergistic effects of *Pds5A* and *Pds5B* deficiencies on enteric nervous system development. (A–C) Whole mount TuJ1 immunofluorescent antibody staining of E12.5 gut highlights the enteric neurons (A, *Pds5A* ^+/−^; B, *Pds5A^−/−^*; C, *Pds5A^−/−^*;*Pds5B^+/−^*). White bars define the ileocecal junction and an asterisk denotes the end of the colon. Enteric neurons colonize the bowel to the mid-colon in wildtype mice (A), but migrate only to the proximal colon or ileocecal junction in *Pds5A^−/−^* (B) and to near the ileocecal junction in *Pds5A^−/−^*;*Pds5B^+/−^* mice (C). Scale bars: 10 µm. (D) Schematic representation of the extent of bowel colonization by enteric neurons *Pds5A^−/−^* mice at E12.5. [WT (*n* = 8), *Pds5A^−/−^* (*n* = 6), and *Pds5A^−/−^*;*Pds5B^+/−^* (n = 3)]. The scale at the top corresponds to the percentage of the respective intestinal segment (small or large intestine) successfully colonized by neurons. (E–G) Whole mount TuJ1 immunofluorescent antibody staining of E11.5 gut highlights the enteric neurons in (E) *Pds5A^+/−^*, (F) *Pds5B^−/−^* and (G) *Pds5A^+/−^*;*Pds5B^−/−^* mutant mice. White bars define the ileocecal junction and an asterisk denotes the end of the colon. Note that the enteric neuron migration wavefront in wildtype mice reaches the ileocecal junction at this age while migration was delayed in *Pds5B^−/−^* mice. In *Pds5A^−/−^*;*Pds5B^+/−^* mice only a few enteric neurons in the stomach were detected and none had migrated into the intestine. Scale bars: 10 µm. (H) Schematic representation of ENS defects in *Pds5A^+/−^;Pds5B^−/−^* mice at E11.5 [WT (*n* = 4), *Pds5B^−/−^* (*n* = 4), *Pds5A^+/−^;Pds5B^−/−^* (*n* = 6)]. The scale at the top corresponds to the percentage of the respective intestinal segment (small or large intestine) successfully colonized by neurons.

*Pds5B*-deficient mice have severe ENS defects, including delayed enteric neuron precursor migration and distal colon aganglionosis [22]. To assess the contribution of PDS5A to ENS development, we performed whole mount staining of *Pds5A^−/−^* fetal gut using TuJ1 antibody, a marker for enteric neurons. By E12.5, enteric neurons had colonized the entire small bowel and half of the colon in wildtype embryos. In contrast, in *Pds5A^−/−^* mice, ENS precursors had failed to migrate much beyond the ileocecal (IC) junction (Fig. 5A and B). To determine the effect of losing three *Pds5* alleles on ENS development we also examined the ENS in E12.5 *Pds5A^−/−^;Pds5B^+/−^* mice and found that they had slightly more extensive distal bowel aganglionosis than *Pds5A^−/−^* mice (Fig. 5A–D) suggesting that there is a *Pds5* gene dosage effect on ENS development. To extend this observation we examined the ENS in *Pds5A^+/−^;Pds5B^−/−^* mice. Because most of these animals die at E11.5, the ENS analysis in these mice was performed at this early stage of development Remarkably, in *Pds5A^+/−^;Pds5B^−/−^* mice (n = 6) we observed very few enteric neurons distal to the stomach, suggesting severe migratory deficits in these mutant ENS precursor cells (Fig. 5E–H). To confirm that heterozygosity for *Pds5A* synergistically exacerbates the ENS defects caused by *Pds5B*-deficiency, we examined *Pds5B^−/−^* animals at this age and found abnormal, but less severe distal bowel aganglionosis. Collectively, these data demonstrate that both PDS5A and PDS5B have important roles in ENS development, but that PDS5B-deficiency causes more severe abnormalities than lack of PDS5A.

### PDS5 proteins regulate lens development

**Figure 6:**
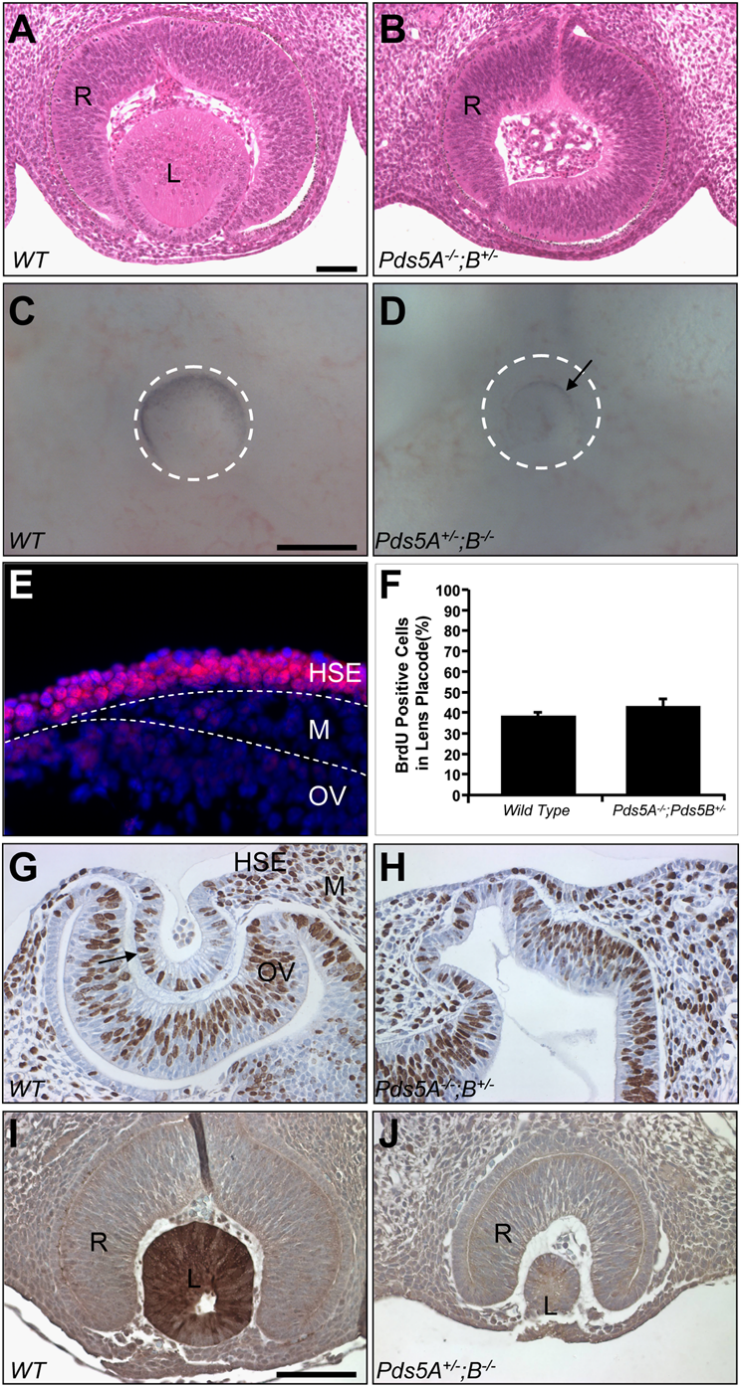
*Pds5A^−/−^;Pds5B^+/−^* and *Pds5A^+/−^;Pds5B^−/−^* mice display abnormal lens development. (A, B) H&E stained sections of eyes from E12.5 wildtype and *Pds5A^−/−^;Pds5B^+/−^* embryos. No lens was formed in the *Pds5A^−/−^;Pds5B^+/−^* embryo (B), whereas the wildtype embryo has a clearly formed lens at this stage (A). (C,D) Whole mount image of the eyes from E11.5 wildtype and *Pds5* mutant embryos. Note that the *Pds5A^+/−^;Pds5B^−/−^* eye (arrow, D) is much smaller than wildtype (white dashed circle, C). (E) Immunofluorescent staining of E9.5 eye using PDS5A antibodies demonstrated that PDS5A is expressed at high levels in head surface ectoderm. HSE, head surface ectoderm; M, mesenchyme; OV, optical vesicle; dashed lines, the boundaries among HSE, M, and OV. (F–H) Proliferating cells in the E10.5 eyes of wildtype (G) and *Pds5A^−/−^;Pds5B^+/−^* (H) embryos labeled with BrdU for 1 hr were detected using anti-BrdU antibodies. The eyes were counterstained with hematoxylin. Eye structures are as indicated in G. Note that the lens placode is invaginating in wildtype (G) but not in *Pds5A^−/−^;Pds5B^+/−^* embryos (H), although the percentage of BrdU positive cells in both wildtype and mutant lens placodes was equivalent (F). (I,J) Immunohistochemical analysis using αA-crystallin antibodies of E11.5 wildtype and *Pds5A^+/−^;Pds5B^−/−^* eyes. Note the small size and lack of αA-crystallin-positive cells in a *Pds5A^+/−^;Pds5B^−/−^* lens (J) compared to strong αA-crystallin expression in the majority of cells in a normal size, wildtype lens (I). R, retina; L, lens.

In analyzing the E11.5 and E12.5 *Pds5A^−/−^;Pds5B^+/−^* and *Pds5A^+/−^;Pds5B^−/−^* embryos, we found a striking defect in lens development, either lens hypoplasia or agenesis (Fig. 6A–D, I, J), that was not observed in *Pds5A*- or *Pds5B*-deficient mice. Interestingly, *Pds5A^−/−^;Pds5B^+/−^* embryos frequently have lens agenesis, whereas *Pds5A^+/−^;Pds5B^−/−^* embryos usually have hypoplastic lenses. An immunohistochemical analysis revealed that PDS5A is highly expressed in head surface ectoderm at E9.5, a stage when the lens is beginning to form (Fig. 6E), whereas PDS5B expression was weak (data not shown). These results suggest that lens formation is more dependent on *Pds5A*, whereas Pds5B plays a more crucial role in ENS development.

To further explore the mechanism of aberrant lens development, we performed a histological examination of E10.5 *Pds5A^−/−^;Pds5B^+/−^* embryos. This revealed diminished contact between the lens placode and optic vesicle (Fig. 6G and H), suggesting that PDS5 proteins are important for this initial contact and the lens induction that follows. Further morphological analysis showed that *Pds5A^−/−^;Pds5B^+/−^* mice had optic vesicles of normal thickness, but with a distorted shape (as seen in Fig. 6H). The peripheral regions of the lens placode were thinner than normal (data not shown) and fewer cells were present in this area. A BrdU labeling experiment however showed that the percentage of proliferating cells in the placode was similar in wildtype and *Pds5A^−/−^;Pds5B^+/−^* mice (Fig. 6F–H). These observations suggest that the abnormal shape of the optic vesicle resulted in decreased contact between it and the surface ectoderm, resulting in a smaller lens placode or failure of lens induction.

The developmental deficits caused by mutations in cohesin are likely due to changes in gene expression. To examine this possibility in the lens, we examined the expression of two transcription factors, *Pax6* and *Sox2*, which regulate lens development. Immunostaining was performed on E9.5 and E10.5 eye sections; however no differences in expression were observed (data not shown). We next examined the expression of αA-crystallin, an abundant lens protein that serves as a marker for lens formation. We found that most cells in the hypoplastic *Pds5A^+/−^;Pds5B^−/−^* lens do not express αA-crystallin (Fig. 6I and J). These results suggest that PDS5 proteins help regulate αA-crystallin expression; however, its loss could also be due to aberrant post-transcriptional regulation or other indirect effects of PDS5 dysfunction. Nonetheless, the lens developmental abnormalities in *Pds5*-deficient mice provide a potential mechanism for how abnormal cohesin activity could cause the myopia associated with CdLS.

### A PDS5B mutation that disrupts DNA-binding contributes to a familial case of CdLS with megacolon

**Figure 7:**
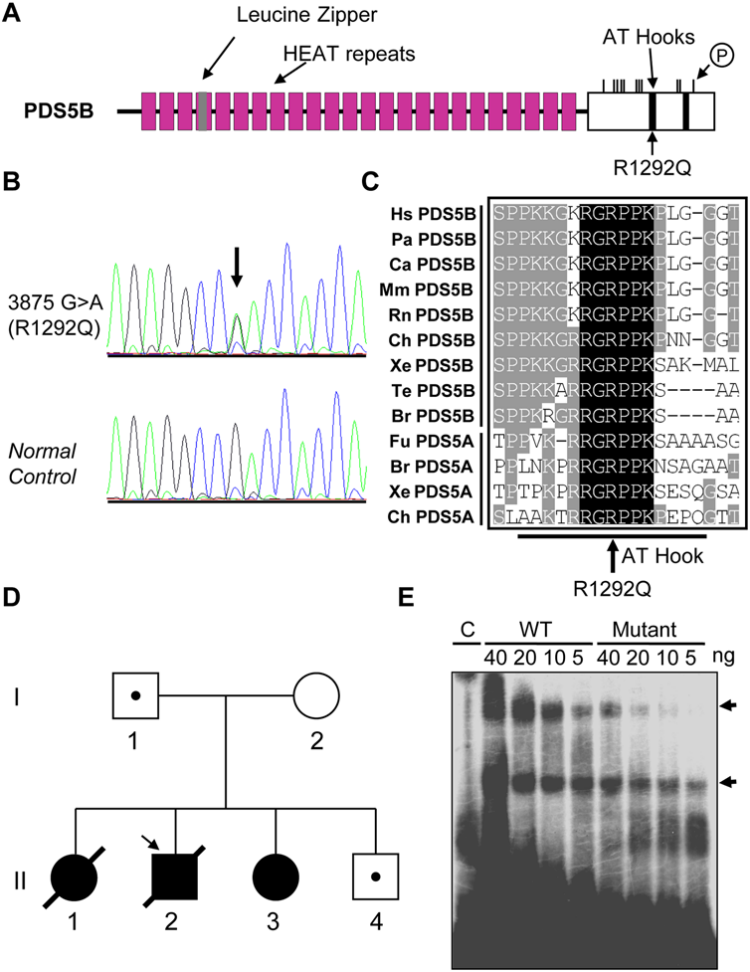
PDS5B is mutated in a case of familial CdLS. (A) A schematic diagram of PDS5B showing the location of the R1292Q mutation within the AT-hook domain. Red rectangles, HEAT repeats; grey rectangle, a leucine zipper domain; blue rectangles, AT-hook domains; thin black bars, phosphorylation sites (Ser, Thr, and Tyr) in the C-terminal region of PDS5B. Note: See additional PDS5B structural analysis in supplementary data (See Fig. S6 in Supplementary Data and Materials S1). (B) Sequencing traces displaying the G to A substitution (highlighted with an arrow) in one *PDS5B* allele in the proband (compare traces of proband and control). This substitution results in an Arg to Gln transition at PDS5B residue 1292. (C) Multispecies alignment of AT-hook domain from PDS5B and PDS5A. Note that the mutated Arg is highly conserved and located within the AT-hook domain. *Hs*, *Homo sapiens*; *Pa*, *Pan troglodytes*; *Ca*, *Canis familiaris*; *Mm*, *Mus musculus*; *Rn*, *Rattus norvegicus*; *Ch*, *Gallus gallus*; *Xe*, *Xenopus tropicalis*; *Br*, *Danio rerio*; *Te*, *Tetraodon nigroviridis*; *Fu*, *Fugu rubripes*. (D) The family tree of a proband (arrow) with three affected siblings (II-1,2,3, blackened circle or square), one clinically normal brother (II-4), and two clinically normal parents (I-1 and I-2). One of the affected siblings was deceased and no DNA was available from this individual (II-1). The father, two affected children, and one unaffected child carry the R1292Q mutation (depicted by blackened symbol or small black dot). (E) The PDS5B(R1292Q) mutant has decreased DNA binding. EMSA using the T5-8-T5 AT-rich probe showed decreased DNA binding by mutant PDS5B compared to wildtype PDS5B. The EMSA was performed with the indicated amounts of PDS5B protein (40, 20, 10, 5 ng). The specific nature of the binding was determined by EMSA competition assays (See Fig. S10 in Supplementary Data and Materials S1). Arrows, protein-DNA complexes. C, control.

Mutations affecting multiple cohesion factors, namely NIPBL, SMC1A, and SMC3 have been identified in CdLS patients, indicating that aberrant cohesin function is responsible for the anomalies associated with this syndrome. The discovery that *Pds5B*- and *Pds5A*-deficient mice exhibit developmental abnormalities resembling CdLS, prompted us to screen patients with CdLS for *PDS5* mutations. We sequenced all exons of *PDS5B* and *PDS5A* and their adjacent intronic sequences that harbor splicing signals in 114 individuals with CdLS that had been screened previously for mutations in *NIPBL*, *SMC1A* and *SMC3* (See Table S2 and S3 in Supplementary Data and Materials S1 for primer information). More than a dozen polymorphisms were detected in both *PDS5A* and *PDS5B* (See Fig. S7 and S8 in Supplementary Data and Materials S1). While no pathologic mutations in *PDS5A* were identified, we found one patient (CDL-238) with a G to A substitution in exon 32 of a single PDS5B allele that creates a missense mutation (R1292Q). This R1292Q substitution occurs at an evolutionarily conserved residue within the invariant core sequence (GRP) of the AT-hook DNA binding motif (Fig. 7A–C). This mutation was not present in 432 control chromosomes. The identification of only a single *PDS5B* mutation in this cohort of CdLS patients suggests that *PDS5B* mutations account for a very small portion of CdLS, a situation similar to that reported for *SMC3* [4].

The parents in the affected family are distantly consanguineous and three of their children were diagnosed with CdLS while one brother and both parents were clinically unaffected (Fig. 7D). We sequenced exon 32 in all available family members and found that two affected siblings (II-2 and II-3) carried the same mutation. In addition, the unaffected brother and the unaffected father also carried the mutation (I-1 and II-4, Fig. 7D). Another affected sibling (II-1) was deceased and no DNA was available. The affected family members showed a common combination of symptoms in the CdLS spectrum, including typical facial dysmorphisms (microbrachycephaly, arched eye brows, synophrys, ptosis, and long philtrum, low posterior hairline, short neck, See Fig. S9 in Supplementary Data and Materials S1), single palmar creases, hirsutism, mental retardation, seizures, and growth retardation (height and weight below the 3^rd^ centiles). The hands were small, but no structural limb abnormalities were noted. In addition, two of the affected siblings (II-2 and II-3) have developed a severe form of megacolon, simlar to that observed in Goldberg-Shprintzen syndrome (OMIM#609460). This family therefore represents a familial CdLS case with atypical inheritance that includes associations with megacolon and mutation of the *PDS5B* gene. To further characterize the *PDS5B* inheritance in this family, we performed SNPs arrays. We found that the two affected siblings (II-2 and II-3) share the same maternal PDS5B allele, whereas the unaffected brother (II-4) inherited a different maternal *PDS5B* allele (See Table S4 in Supplementary Data and Materials S1). This result suggests that CdLS is transmitted in a recessive manner in this family and that a second mutation is likely to be present in the maternal *PDS5B* allele that was not detected by our sequence analysis.

The location of the R1292Q mutation within the AT-hook domain in PDS5B suggests that it could be functionally significant. The positively charged Arg within the core sequence of the AT-hook domain in high mobility group (HMG) proteins has been demonstrated by structural analysis and electrophoretic mobility shift assays (EMSA) to be crucial for DNA binding [39], [40]. To test the effect of the R1292Q mutation on PDS5B function, we performed EMSA using the C-terminal region containing the AT-hook domains from both wildtype and R1292Q mutant PDS5B. As reported for the AT-hook containing HMG-1, we found that the PDS5B C-terminal region bound to the AT-rich DNA probe efficiently and specifically. However, the C-terminal region derived from PDS5B (R1292Q) had decreased DNA binding affinity (Fig. 7E and See Fig. S10 in Supplementary Data and Materials S1). Interestingly, we observed two DNA-protein complex bands on our EMSA assays (Fig. 7E and See Fig. S10 in Supplementary Data and Materials S1), suggesting that the two AT-hook motifs in PDS5B imbue it with multivalent binding properties like that of HMG-1 [41]. While it has been previously speculated but never demonstrated that PDS5 can bind DNA [42], these results indicate that the R1292Q mutation disrupts the DNA binding affinity of the C-terminal PDS5B AT-hook domains. This presumably influences PDS5B-mediated transcriptional regulation, and may affect cohesin activity and development thus resulting in CdLS.

## Discussion

The cohesin complex mediates sister chromatid cohesion during cell division, regulates gene expression, and influences a broad spectrum of prenatal and postnatal developmental processes. In humans, subtle perturbations in cohesin activity contribute to disorders now known as ‘cohesinopathies’, such as CdLS and RBS, which are caused by mutations in *NIPBL*, *SMC1A*, *SMC3*, and *ESCO2*. While ∼50% of CdLS patients have identifiable mutations in *NIPBL*, mutations in other cohesion components (such as *SMC1A* and *SMC3*) appear to be much rarer (∼5%), leaving many clinically defined CdLS patients with no identified genetic lesion [4]. We previously reported that mice deficient in *Pds5B* exhibit a spectrum of developmental defects reminiscent of those present in CdLS.

In this study, we sequenced the *PDS5A* and *PDS5B* genes in a cohort of CdLS patients and identified a mutation in a conserved motif (AT-hook domain) in PDS5B, which likely causes or contributes to the disease. Our analysis of PDS5B has identified two AT-hook domains [22] and a degenerative AT-hook domain in PDS5A (See Fig. S6 in Supplementary Data and Materials S1). We demonstrated that the PDS5B C-terminal region binds to an AT-rich DNA sequence motif similar to that recognized by HMG-1 [41], and that the CdLS-associated mutation in PDS5B at R1292Q decreases PDS5B DNA-binding affinity. The association of PDS5 with chromatin is dependent on functional cohesin in Hela cells and *Xenopus* egg extracts [30], suggesting that the PDS5B-DNA interactions demonstrated here may not be sufficient for recruitment of PDS5B to chromatin *in vivo*. Instead, PDS5B could interact with the cohesin core complex and modulate the subchromatin distribution of chromatin-loaded cohesin through its AT-hook domains. The acquisition of AT-hook domains in both PDS5A and PDS5B during evolution may therefore reflect the predilection of cohesin to interact with heterochromatin (i.e., AT-rich) chromosomal regions. The AT-hook motif can also interact with scaffold associated regions (SARs) [43], [44] that can act as *cis* regulatory elements for SATB1 and SATB2, transcription factors that regulate skeletal patterning and palatogenesis [45], [46].

The functional mutation of *PDS5B* we identified in a familial CdLS case with atypical inheritance pattern is strongly suspected to be pathogenic, although further studies using mice expressing this mutant protein will be important in confirming its role in this syndrome. The occurrence of the mutation in unaffected family members has been previously demonstrated in one familial case of CdLS with an *SMC1A* mutation [8] and could be explained by three possibilities. First, the identification of a functional mutation in *PDS5B* and the segregation of maternal alleles in a disease-specific manner, as well as the recessive nature of the deficits in *Pds5B* mutant mice suggest that CdLS in this family is caused by compound heterozygosity of *PDS5B* mutations. In this case, the second *PDS5B* mutation could be a point mutation in an intron or in the regulatory region of *PDS5B* or other rearrangements like a deletion or translocation that would not be detected by sequencing.

A second possibility for the atypical genetic inheritance of *PDS5B* mutation in this family is dominant inheritance with incomplete penetrance. This is consistent with the variable expressivity and penetrance observed in *Pds5B*-deficient mice, suggesting that modifier genes or epigenetic factors contribute significantly to PDS5B-regulated development. Genetic or epigenetic modifers may be common in CdLS as there is significant phenotypic variability among patients with the same mutation, even within families [9]–[11]. In addition, the consanguinity of the parents may provide recessive inheritance of a modifier locus (or loci) whose homozygosity produces an environment whereby a single mutant *PDS5B* allele leads to developmental deficits.

Finally, it is possible that this represents a digenic pattern of inheritance. In this case, the other mutation could be present in other cohesin proteins that regulate *PDS5B* function. An example of such an inheritance pattern is Bardet-Biedl syndrome (BBS), a rare oligogenic disorder exhibiting genetic heterogeneity with primary cilium dysfunction [47]. Like CdLS, the BBS phenotype is variable both between and within families. Interestingly, in some cases of BBS, mutations in two cilium genes are required for phenotypic penetrance [48]. A similar situation exists for Hirschsprung disease, where hypomorphic mutations in both RET and endothelin can interact to cause the characteristic ENS deficits normally associated with heterozygous loss-of-function mutations in these genes [49].

Perhaps the most consistent and striking abnormalities in *Pds5* mutant mice concern the aberrant migration of ENS precursors that results in intestinal hypoaganglionosis. This is not reported as part of the constellation of symptoms in CdLS, but could be related to the chronic constipation that is present in these patients [50]. However, in this particular case these observations take on added interest as affected members of this family also develop severe megacolon. This disorder is similar to Hirschsprung disease, which is most often caused by mutations in the RET tyrosine kinase [51]. These symptoms are consistent with the distal colon hypoganglionosis observed in *Pds5B*-deficient mice. This condition becomes more severe with decreasing *Pds5* gene dosage, at which point it closely resembles deficits observed in *Ret* mutant mice [52]. Interestingly, *Ret*-deficient mice also have deficits in sympathetic neurons that are similar to those present in *Pds5B*-deficient mice. Furthermore, cohesin mutations in zebrafish block expression of RUNX1, a transcription factor that regulates *Ret* expression in mammals [20]. These findings suggest that *Pds5B* and *Ret* may act on similar pathways to regulate neuronal development. Indeed, the identification of PDS5B mutations in a family with CdLS and megacolon suggests that PDS5B could be a modifier for Hirschsprung disease and, that these two disorders may share additional molecular pathways.

Cohesin functions along with the chromatin insulator protein CTCF to regulate long-range enhancer-promoter communication-mediated gene expression, possibly through stabilizing CTCF-mediated chromosomal loops [53]. These long-range effects on gene expression may be altered by defective cohesin function and ultimately be responsible for the developmental abnormalities present in CdLS and RBS. Cohesin and CTCF binding occurs at loci containing the *Dlx5* and *Bdnf* genes [23], which encode proteins crucial for development of the skeleton and nervous system. Interestingly, cohesin and CTCF are bound to the *Bdnf* locus at several sites, including the promoter for isoform III, which is regulated by MeCP2 in a neuronal activity-dependent manner. Furthermore, the MeCP2-mediated expression of *Dlx5* is dependent on changes in chromosomal looping [54], suggesting a potential mechanism by which mutations in cohesin components (e.g., in CdLS) could be translated into aberrant neuronal circuitry.

The *Pds5A* mutant mice have many developmental defects that are similar to those present in *Pds5B*-deficient mice and in humans with CdLS. Other phenotypes that are associated with *Pds5B*-deficient mice, like defects in the ENS discussed above as well as autonomic nervous system and primordial germ cell abnormalities were not present (or as severe) in *Pds5A*-deficient mice, suggesting both redundancy and/or diversification of the roles of PDS5 proteins during evolution. The broad spectrum of abnormalities with variable expressivity in *Pds5A*- and *Pds5B*-deficient mice is typical of CdLS [1], suggesting genetic, epigenetic or environmental factors can influence the effects of cohesin dysfunction. The striking phenotypes seen in these mice also suggest that additional human disorders with similar phenotypic overlap could be caused by mutations in PDS5A or PDS5B. Interestingly, the number of human diseases caused by mutations in cohesion proteins (cohesinopathies: CdLS, RBS/SC phocomelia, and Alpha thalassemia/mental retardation X linked (ATRX))[2], [5], [55] is growing. The *Pds5A*- and *Pds5B*-deficient mice may be useful for the identification of genetic modifiers and genetic pathways critical for cohesin-regulated development that would constitute additional targets for mutational analysis in cohesinopathies.

Our inability to detect *Pds5A;Pds5B* double homozygous embryos indicates that PDS5 function is essential for early embryogenesis and may reflect its importance in mitosis or sister chromatid cohesion. While loss of either *Pds5A* or *Pds5B* caused neonatal lethality, the loss of three *Pds5* alleles leads to mid-gestational embryonic death. Although these embryos showed striking defects in the enteric nervous system, heart, and lens, cells with only a single remaining *Pds5* allele did not manifest precocious sister chromatid separation. These results suggest that a low level of PDS5 protein is sufficient for chromosome cohesion but is unable to sustain cohesin-mediated developmental gene expression. The differential sensitivities of developmental gene regulation and chromosome cohesion on cohesin dosage are corroborated by observations in other model organisms. In *Drosophila*, heterozygous mutants of either *Nipped-B* or *Pds5* have decreased expression of the homeobox *cut* gene without cohesion abnormalities, whereas cohesion defects are present in the homozygous mutants [21]. In zebrafish reduced cohesin levels lead to abnormal *Runx1* expression, indicating that cohesin levels affect transcriptional regulation [20]. Finally, in humans, heterozygous mutations in *NIPBL*, *SMC1A*, or *SMC3* lead to CdLS, which is characterized by multiple developmental anomalies without overt cohesion defects [4]. Although consistent with the hypothesis that perturbed non-canonical functions of cohesin are responsible for abnormalities present in cohesinopathies [2], [3], [56], the lack of cohesion defects in cells with low levels of PDS5 proteins is in contrast to PDS5 functions in budding yeast, *C. elegans*, and *Drosophila*. These organisms each contain a single PDS5 protein, and it plays a critical role in chromosome segregation [13], [21], [57]. It should be noted, however, that only mild cohesion defects were found in Hela cells when either *PDS5A* or *PDS5B* were knocked down by RNAi [30]; again, suggesting that low amounts of PDS5 protein are sufficient to support cohesion.

In summary, we have discovered that PDS5B is mutated in a familial case of CdLS with megacolon, and that PDS5A and PDS5B have both redundant and distinct roles in development that are likely unrelated to its ancient function in sister chromatid cohesion. The *Pds5A*- and *Pds5B*-deficient mice provide valuable mammalian models to study molecular mechanisms of developmental functions of the cohesin complex and the pathogenesis of cohesinopathies.

## Materials and Methods

### Ethics Statement

All subjects enrolled in this study were consented under an IRB-approved protocol of informed consent through The Children's Hospital of Philadelphia. All consents were written and signed by the participants or by their parents or guardians in the case of minors. All mouse experiments were performed under protocols approved by the Washington University Animal Studies Committee.

### Re-sequencing of PDS5B and PDS5A

All subjects were evaluated by one or more clinical dysmorphologists with experience with CdLS patients (supervised by Ian Krantz, U. Penn). All of these samples were pre-screened by investigators at U. Penn and found to have no mutations in *NIPBL*, *SMC1A*, and *SMC3* [2], [4], thus they have unidentified genetic alterations.

Using primer design software (Primer3), amplification primers for each exon of *PDS5B* (36 exons) and *PDS5A* (33 exons) were designed so that the intron-exon junctions as well as the entire exon were sequenced in both directions (Tables S1 and S2)). Forward primers contain M13 forward sequence at the 5′ end and reverse primers contain M13 reverse sequence at the 5′ end so that each amplicon could be sequenced with the universal M13 forward and reverse sequencing primers. Primers were ‘validated’ in a trial PCR reaction using control DNA to ensure that a single fragment of predicted size was obtained. If a primer set failed to amplify the anticipated product, new primers were designed for the failed amplicon, and the process was repeated until specific amplicons were obtained. The sequencing traces were analyzed in Mutation Viewer (MV, bioinformatics core of Washington University in St. Louis), and ‘high confidence’ sequence alterations that did not coincide to polymorphisms present in SNP databases were verified by examining the sequence traces directly. Alterations that were clearly present were confirmed by re-amplifying and sequencing the suspect amplicons.

### Purification of Recombinant C-terminal PDS5B and Electrophoretic Mobility Shift Assays

C-terminal human PDS5B (a.a. sequence: 1134–1446, either wildtype or R1292Q mutant) was amplified by PCR using primers (forward primer: 5′-GCTAGTCATATGTACCCGTACGACGTCCCGGACTACGCTTCTGAATTGGAGAAGCCTAGAGGCA-3′ and reverse primer: 5′-GCTAGTAAGCTTTCGCCGTTCCCTTTTAGCACT-3′) and an N-terminal HA tag was introduced. This fragment was cloned into the *pET30a(+)* vector so that it also contained a C-terminal 6xHis tag. BL21 Rosetta cells (Novagen) were transformed with this HA and 6xHis tagged C-terminal PDS5B *pET30a(+)* vector and grown to mid-log phase (OD_600_ = 0.6). Protein expression was induced by addition of isopropyl-β-D-thiogalactopyranoside (final conc. = 150 µM) and the bacterial cells were lysed 2 hr later using the cell lytic kit (Sigma). His-tagged PDS5B fusion proteins were purified on nickel-nitrilotriacetic acid columns (Sigma). Eluted proteins were concentrated using Amicon columns (Millipore), and the concentration was determined using the BCA protein assays (Pierce), equalized by dilution, and dialyzed against Buffer A (50 mM Tris, pH 7.5, 0.5 mM EDTA, 100 mM NaCl, 10% glycerol, 1 mM dithiothreitol) overnight. Electrophoretic Mobility Shift Assays (EMSA) were performed as previously described [41]. Briefly, 20 ng of purified protein was mixed with 2.25 pmol of ^32^P-labeled double-stranded oligonucleotide probe (∼30,000 cpm, T5-8- T5) and incubated at 25°C for 5 min. The mixture was electrophoresed through a 6% polyacrylamide gel and the shifted fragments were visualized by autoradiography. Sequences of the oligonucleotides used to make the T5-8-T5 DNA probes are: 5′-GGACTCCAGGTCCAGGACCGCTTTTTGCGCGCGCTTTTTGCGGGAGGTCCAGCTGTCCACCTCC-3′ and 5′-GGAGGTGGACAGCTGGACCTCCCGCAAAAAGCGCGCGCAAAAAGCGGTCCTGGACCTGGAGTCC-3′.

### Immunohistochemistry and Western blot analysis

Tissues used in immunohistochemistry were fixed in paraformaldehyde/Bouin's fixative. For antigen retrieval, paraffin-embedded sections were de-paraffinized in xylene, hydrated and boiled in 1 mM EDTA solution for 30 min prior to immunostaining [22]. Primary antibodies used in this study include sheep anti-tyrosine hydroxylase (1∶200; Chemicon), anti-αA-crystallin (1∶500; antibody kindly provided by Usha Andley from hybridoma cells isolated by Paul FitzGerald), and rabbit anti-PDS5A (1∶1,000; Bethyl Laboratories, Montgomery, TX). The signals were visualized using donkey anti-sheep HRP (1∶200; Jackson immunoresearch), donkey anti-mouse HRP (1∶100, Jackson immunoresearch), or goat anti-rabbit Cy3 (1∶500; Jackson ImmunoResearch) secondary antibodies.

Western blot analysis was performed using standard techniques and proteins derived from E18.5 kidney. The kidneys were homogenized in 2× SDS protein lysis buffer (100 mM Tris-HCl, pH 6.8, 4% SDS, 1× protease inhibitor cocktail (Roche)) and 40 µg of total protein was loaded onto the gel. The blots were probed with rabbit anti-PDS5A antibody (1∶10,000 dilution; Bethyl Laboratories, Montgomery, TX), mouse monoclonal anti-β-galactosidase (1∶1000 dilution, from Roche), or rabbit anti-SMC3 antibody (1∶1000 dilution; kindly provided by Rolf Jessberger from Dresden University of Technology). The signals were visualized using species-specific HRP conjugated secondary antibodies and chemiluminescence substrate (Pierce). For a loading control, mouse anti-β-tubulin antibody (DSHB, University of Iowa, IA) was also used at 1∶20,000 dilution.

### Histological analysis of bone and palate

Alizarin Red S and Alcian Blue staining of newborn mice was performed as previously described [22]. Briefly, newborn mice were de-skinned and dehydrated in 95% EtOH. They were then stained in Alcian blue solution for 3 d, rehydrated and incubated in 1% KOH for 2 d, and stained for Alizarin Red S for 3 d. For embryonic and newborn heads, fixation was carried out in 4% paraformaldehyde at 4°C for 16 h. The fixed heads were properly oriented in paraffin and coronal sections were prepared. The sections were stained with Hematoxylin and Eosin (H & E) and examined microscopically for evidence of cleft palate.

### Analysis of germ cells, testes transplantation, and the renal system

Embryonic gonads were dissected, fixed in Bouin's solution overnight at 4°C, embedded in paraffin, and 6 µm sections were prepared. Germ cells were examined by H&E staining.

Testes from E18.5 mutant and *wild-type* mice were transplanted subcutaneously onto the back/flank of castrated 4- to 8-wk-old male nude mice (Taconic #NCRNUM, Germantown, NY) as previously described [58]. The grafted donor testes were harvested and processed for histological evaluation at the indicated time points. To determine relative nephron numbers, we serially sectioned E18.5 kidneys in their entirety and counted glomeruli every 120 µm [52].

### Analysis of cardiac malformations

The E12.5, E18.5, or newborn thorax was fixed in 4% formaldehyde before the entire thorax or dissected heart was embedded in paraffin. The tissue blocks were serially sectioned completely at 6 µm thickness, hematoxylin and eosin stained, and inspected for defects [22].

### Nervous system analysis of *Pds5* mutant mice

Dissociated hippocampal neurons from E15 mouse embryos were cultured in Neurobasal medium with B27 (Invitrogen). The anti-mitotic reagent, 5-Fluoro-2′-deoxyuridine (FDU) (Sigma #F0503), was used at a final concentration of 20 µM for the first three days of culture to eliminate proliferating, nonneuronal cells. To study polarity, neuronal morphology was analyzed at 3 DIV by immunostaining with neuronal-specific beta III tubulin (TuJ1) (Covance) antibody. Neurons bearing only a single long axon, indicative of normal neuronal polarization, were counted. The neurons were stained with tau-1 antibody to confirm the neuronal processes were axons.

For analysis of the enteric nervous system, E11.5 or E12.5 gut was dissected from the mouse, fixed with 4% paraformaldehyde at 25°C for 30 min, and stained as a whole mount with TuJ1 antibody. The sympathetic nervous system was analyzed using whole-mount TOH immunohistochemistry as described previously [22].

### Analysis of lens development

The heads of E9.5 to E12.5 embryos were fixed in 4% paraformaldehyde and embedded in paraffin with a specified orientation for all specimens. Serial sections were stained with H & E for microscopic examination. For BrdU immunostaining, timed pregnant females were injected with 50 mg/kg body weight of a mixture of 10 mM BrdU (Roche, Indianapolis, IN) and 1 mM 5-fluoro-5-deoxyuridine (Sigma, St. Louis, MO) and sacrificed after 1 hour. Paraffin-embedded sections of embryonic head were incubated with a monoclonal anti-BrdU antibody (1∶250, Dako, Carpinteria, CA) and visualized with a Vectastain Elite Mouse IgG ABC kit (Dako, Carpinteria, CA).

### Metaphase spread analysis of mouse embryonic fibroblast cells

Chromosome analysis was performed in the cytogenetics core at Washington University Medical School. Mouse embryonic fibroblasts from *wildtype*, *Pds5A*, *Pds5A^+/−^;Pds5B^−/−^*, *Pds5A^−/−^;Pds5B^+/−^ or Pds5B* mutant mice (passage 1–3) were cultured in DMEM with 10% fetal bovine serum. Chromosomes were harvested the day after plating after growth in colcemid (1 ug/ml) for 1 h using standard hypotonic (0.075 M KCl) treatment and fixation in methanol-acetic acid (3∶1). Slides were prepared and chromosomes were analyzed using the GTG (giemsa-banding using trypsin and giemsa) banding method. More than two cell lines were analyzed for each genotype. More than two cells were fully analyzed from each line, and all were found to have normal karyotypes. More than fifty cells were examined for precocious sister chromatid separation (PSCS) for each line.

### Fluorescence recovery after photobleaching (FRAP) analysis of SCC1-EGFP

The SCC1 cDNA was released from pEGFP-N1-SCC1 (gift from Cryns VL; [17]) by digestion with *Bgl*II and *Age*I and inserted into the *Bam*HI and *Age*I sites of FUGW (gift from Baltimore D; [59]) to generate the SCC1-EGFP fusion construct in lentivirus vector (named FU-SCC1-EGFP). Lentivirus expressing SCC1-EGFP was made by standard methods with a titer of 10^6^ to 10^7^ infectious particles per mL. MEF cells were infected with lentivirus for 12 hr followed by two washes with medium to remove remaining virus. Three days after infection, MEF cells were cultured on Lab-Tek Chambered CoverGlasses slides (Nunc) with CO_2_-independent medium (Invitrogen) for FRAP. Half of the nucleus was repeatedly bleached using a Leica SP5 confocal microscope equipped with a 60×1.4 oil immersion objective at high laser power with a 6–8× zoom. The fluorescence intensity was measured every 30 sec for 30 min post-bleaching with the laser on low power. The difference in fluorescence between the unbleached and bleached regions over time was calculated to generate the re-equilibration curve. The fraction of weak vs. strong chromatin bound SCC1-EGFP as well as their residence times were calculated using bi-exponential function fitting as described [35]. Unbound/soluble and chromatin-bound fractions were determined by comparing the SCC1-EGFP fluorescences of unbleached half nuclei before photobleaching and at t = 0 s after photobleaching, when nuclear-localized soluble EGFP that cannot bind chromosomes gets completely bleached (Dorsett D, personal communication). Re-equilibration curves incorporating data from t = 0 s after photobleaching and bi-exponential function fitting contribute to the detection of weak chromatin-bound SCC1 component, which has not been detected before [35]. MEFs cannot be synchronized, so we were unable to detect the differences in SCC1-chromatin association dynamics in G1 vs. G2 cells in these FRAP assays.

### Statistics

Data are expressed as mean±SD. Student's *t* test was used for comparisons between groups. *P* values of less than 0.05 were considered significant.

## Supporting Information

Supplementary Data and Materials S1 (2.15 MB PDF)
