# Correction: Dosage Effects of Cohesin Regulatory Factor PDS5 on Mammalian Development: Implications for Cohesinopathies

**DOI:** 10.1371/annotation/ea5b7eb5-5087-448a-8325-c8efff1f54d9

**Published:** 2009-05-19

**Authors:** Bin Zhang, Jufang Chang, Ming Fu, Jie Huang, Rakesh Kashyap, Ezequiel Salavaggione, Sanjay Jain, Shashikant Kulkarni, Matthew A. Deardorff, Maria L. Giovannucci Uzielli, Dale Dorsett, David C. Beebe, Patrick Y. Jay, Robert O. Heuckeroth, Ian Krantz, Jeffrey Milbrandt

The eighth author's name is incorrect. The correct name is: Shashikant Kulkarni. 

