# Supplementary material for: Dosage Effects of Cohesin Regulatory Factor PDS5 on Mammalian Development: Implications for Cohesinopathies
Source: PLoS One. 2009 May 1;4(5):e5232. doi: 10.1371/journal.pone.0005232 (PMC2672303; doi:10.1371/journal.pone.0005232)
Supplement: Supplementary Data and Materials S1 — (2.15 MB PDF) [file pone.0005232.s001.pdf]

Supplementary data

Generation of *Pds5A*-deficient mice and the expression pattern of *Pds5A*.

To test whether PDS5A, the paralogue of PDS5B, can compensate for loss of PDS5B and, to define its physiological roles, we generated mice deficient for *Pds5A*. We identified an embryonic stem cell line carrying a gene-trap allele for *Pds5A* in the Baygenomics database (cell line ID: *RRM243*; http://www.baygenomics.ucsf.edu). RT-PCR and nucleotide sequencing were performed to verify the hybrid mRNA message generated by splicing between *Pds5A* exon 2 and the β-geo insertion (Fig. S1A; sequencing data not shown). This indicated that the *pGT0lxf* vector is inserted within intron 2 and disrupts the normal splicing of *Pds5A* gene (i.e., downstream of *Pds5A* exon 2). Southern blot analysis with a *NEO* probe indicated that only one copy of the gene trap vector was inserted into the genome in RRM243 ES cells (Fig. S1B). To determine the exact insertion site and to facilitate future genotyping, we modified splinkerette PCR [1] and determined the *pGT0lxf-Pds5A* intron junction at the nucleotide level (Fig. S1C). We detected the *Pds5A* gene-trap allele (designated as $Pds5A^{-}$) by PCR (Fig. S1D) and Southern blot analysis (data not shown). We mated mice heterozygous for the gene trap allele ($Pds5A^{+/-}$) and found that *Pds5A* homozygous mice died perinatally (within 24 hr of birth) (data not shown). We demonstrated that the gene trap allele was a *Pds5A* null allele by Western analysis using anti-PDS5A antibodies. PDS5A is not detectable in E18.5 homozygous mutant kidneys. A PDS5A-β-galactosidase fusion protein containing sequences from the *Pds5A* exon 2 was detected in both $Pds5A^{+/-}$ and $Pds5A^{-/-}$ kidneys (Fig. S1E). Expression analysis using qRT-PCR showed expression of PDS5A in most tissues. β-galactosidase staining demonstrated that PDS5A was expressed in Purkinje cells in the cerebellum, neurons in the cortex and retina, in the peripheral region of the seminiferous tubules, epithelial cells in renal tubules, and cells in the thymic

medulla (Fig. S3). High levels of PDS5A were also detected in cultured dorsal root ganglion and hippocampal neurons (data not shown).

Functional conservation and diversification of PDS5A and PDS5B during evolution.

To examine the evolutionary conservation of PDS5 proteins, we generated a phylogenetic tree using PDS5 protein sequences from many species (Fig. S6). While there are two PDS5 homologues in mammals, only one PDS5 protein is present in lower organisms, like fungi, flies, and worms. PDS5A and PDS5B in vertebrates share high similarity in the N-terminus (1-1140: 77% identity and 88% similarity) but diverge substantially at the C-terminus (1141-1360: 17% identity and 29% similarity). The N-termini of PDS5A and PDS5B each contains 23 HEAT repeats and one leucine zipper domain ($LX_6LX_6LX_6L$), motifs commonly associated with protein-protein interactions (Fig. S6). Several other cohesion components also contain HEAT repeats, including SCC2, SCC3, and WAPL [2, 3].

We previously identified two HMG-I(Y)/AT-hook domains in the carboxy terminus of mammalian PDS5B, but failed to recognize these motifs in PDS5A [4]. The AT-hook domain was initially identified in the HMG-I/Y family and imbues these proteins with the ability to bind the minor groove of A-T tracts [5]. New analyses have widened the range of AT-hook sequences, thus we re-visited the question of whether PDS5A contains this motif. We performed multi-species alignment of C-terminal regions of PDS5A and PDS5B and found that PDS5A proteins from fish and chicken contain classical AT hook domains (Fig. S1), whereas mammalian PDS5A proteins contain a single degenerative AT-hook domain variant (Fig. S6) [6]. So far, no other functional DNA binding domains have been identified in cohesin components even though cohesin is tightly associated with chromosomes. While cohesin forms a ring structure to trap sister chromatids [7], genome-wide mapping of cohesin sites indicates that cohesin is

preferentially located near AT-rich sequences in yeast [8] and is colocalized with CTCF in humans [9]. AT-rich sequences are commonly found in heterochromatin regions and scaffold associated regions (SAR) [10, 11], sites to which cohesin is bound. Thus, AT-hook domains in PDS5A and PDS5B may contribute to the spatial distribution of cohesin on chromosomes.

Supplementary methods

Generation of *Pds5A*-deficient mice and mapping the genomic insertion of β-geo into the *Pds5A*$^-$ locus.

One ES cell clone (RRM243, Baygenomics) was injected into blastocysts by standard methods to generate chimeric mice. The chimeras were mated and successful germline transmission of the mutant *Pds5A* allele was detected by PCR. All experiments were performed on littermates with a mixed 129Sv/129Ola/B6 background (usually F1 or F2 generation) under protocols approved by the Washington University Animal Studies Committee.

We performed Southern blot analysis on genomic DNA derived from ES cells digested with either *Pst*I, *Bgl*II, *EcoR*V, *Nco*I, or *Ase*I restriction enzymes. The blot was probed with a probe from the neomycin resistance cassette (NEO probe, sequence available upon request) to determine the number of inserted *pGT0Lxf* vector copies. The method to map the gene-trap cassette insertion site was modified from the Splinkerette PCR assay described previously [1]. Briefly, genomic DNAs from mouse tails of β-*geo* positive offspring from the RRM243 chimeras were digested with *Bfa*I at concentration of 100 ng/ul. Digested genomic DNAs were ligated with splinkerette linkers for 1 hr followed by treatment with *Bfa*I for 1 hr. Splinkerette linkers were made by annealing equimolar amounts of the primerette-long (5’-GATCCTCCACTACGACTCACTGAAGGGCAAGCAGTCCTAACAAG-3’) and the splink

(5'-TACTTGTTAGGACTGCTTGGAGGGGAAATCAATCCCCT-3'). Primary PCR was performed using the primerette-short primer (5'-GATCCTCCACTACGACTCACTGAAGGGC-3') and gene-trap vector primary primer (5'-GCTACAGGCATCGTGGTGTCACGCTCGT-3') for 10 cycles of 95 ^oC for 5 sec and 70 ^oC (-0.5 ^oC/cycle) for 2 min followed by 20 cycles of 95 ^oC for 5 sec and 65 ^oC for 2 min. Nested PCR was performed using 1/250 diluted primary PCR product with primerette-nested primer (5'-GGGCAAGCAGTCCTAACAAGTA-3') and gene-trap-vector-nested primer (5'-GCAGTGTTATCACTCATGGTTATGGCA-3') for 30 cycles of 95 ^oC for 5 sec, 61 ^oC for 30 sec, and 70 ^oC for 90 sec. The PCR products were purified by electrophoresis and extracted using Zymoclean gel DNA recovery kit (Zymo Research, Orange, CA) and sequenced with the primers used above (primerette-nested primer and gene-trap vector-nested primer).

The genotypes of *Pds5A*$^-$ mutant mice were determined by PCR using primers P1 (5'-CCATCCTCTCATTCATAATCCATTTAACA-3'), P2 (5'-CCAACTTGGAGGGAAGGGTTTAGCTTACA-3'), P3 (5'-GTGAGTACTCAACCAAGTCATTCTGAGA-3') (95°C for 30 sec, 58°C for 60 sec, 72°C for 90 sec; 35 cycles). Wildtype and mutant alleles were amplified with P1-P2 (432 nt amplicon) and P1-P3 (293 nt amplicon) primer pairs, respectively.

Quantitative RT-PCR

RNA was prepared from mouse adult and embryonic tissues using Trizol (Invitrogen) and quantified by the Ribogreen fluorometric assay (Molecular Probes). Reverse transcription was performed using M-MLV reverse transcriptase (Invitrogen) and oligo dT and random hexamers as primers. qRT-PCR was performed using a Model 7700 instrument (Applied Biosystems) using *Sybr* Green I fluorescence (Molecular Probes) as described [4]. Target genes were

analyzed using standard curves to determine relative levels of gene expression. Individual RNA samples were normalized according to the levels of GAPDH or 18S mRNA [4]. qRT-PCR for *Pds5A* was performed using forward primer: 5'-GCTCAAAAATCTGTGCCAGCT-3' and reverse primer: 5'-CTGACCTTCCCCAGATACGG-3'.

Phylogenetic analysis of PDS5

The phylogenetic tree of PDS5 was constructed based on a previously described method [12, http://www.treefam.org]. The PDS5 family was defined as a group of genes evolved from a single gene in the last common ancestor of all animals. Briefly, this method creates a seed family for PDS5 using related animal and fungal PDS5 from the PhIGs database, and expands to full family of PDS5 using BLAST and HMMER database searching. The protein sequences of the full PDS5 family were aligned and the alignment was then filtered to keep only conserved regions. Using the filtered alignments as input in the neighbor-joining algorithm, a phylogenetic tree of PDS5 was constructed based on amino acid mismatch distances. The tree was bootstrapped 100 times and visualized by Treeview (http://taxonomy.zoology.gla.ac.uk/rod/treeview.html).

HEAT repeats analysis

Yeast PDS5 contains HEAT repeats at the N-terminus [13]. To identify the sequence and number of HEAT repeats in mammalian PDS5 proteins, we analyzed human PDS5A and PDS5B using secondary structure predictions and disorder probability calculated by the PhD program (http://cubic.bioc.columbia.edu/predictprotein/). The predicted secondary structure composition for PDS5B showed about 54% alpha helical content, 1% beta-structure/strand, and 45% loop. In the region containing the first 1100 PDS5B residues, approximately every 40 residues there were two peaks of high disorder probability with a smaller peak in the center of the region. A hidden

Markov model (HMM) of the HEAT-repeat domain [14] predicted positive scores (>1000) in 15 fragments and marginal scores in 8 fragments. We thus delineated 23 HEAT repeats in PDS5A and in PDS5B. This HEAT repeat analysis for PDS5A and PDS5B is consistent with a previous analysis of yeast PDS5 [13].

β-galactosidase staining

Freshly dissected tissues or embryos were fixed in 0.1 M phosphate buffer (pH 7.2) with 0.2% glutaraldehyde, 5 mM EGTA, 2 mM MgCl2, 0.1% NP-40, for 30 min at 4 ^{o}C. Ten-micrometer sections of the sucrose cryoprotected tissues were cut on a cryostat and thaw-mounted on Superfrost/Plus slides (Fisher). Bacterial β-galactosidase histochemistry was performed as previously described [15] in a reaction solution containing 1 mg/ml X-gal, 5 mM potassium ferrocyanide, 5 mM potassium ferricyanide, 0.02% NP40, 0.01% sodium deoxycholate, and 2 mM $MgCl_2$ in 0.1 M phosphate buffer (pH 7.2). After staining for 12-16 hr, slides were refixed in 4% paraformaldehyde in 1 X PBS (pH 7.3) for 15 min at 25 ^{o}C. Slides were counterstained with nuclear fast red and examined by light microscopy. All tissues were examined with wildtype controls to ensure specificity staining.

Supplementary References

1. Dupuy, A.J., Fritz, S., and Largaespada, D.A. (2001). Transposition and gene disruption in the male germline of the mouse. Genesis *30*, 82-88.
2. Kueng, S., Hegemann, B., Peters, B.H., Lipp, J.J., Schleiffer, A., Mechtler, K., and Peters, J.M. (2006). Wapl controls the dynamic association of cohesin with chromatin. Cell *127*, 955-967.

3. Neuwald, A.F., and Hirano, T. (2000). HEAT repeats associated with condensins, cohesins, and other complexes involved in chromosome-related functions. Genome Res *10*, 1445-1452.

4. Zhang, B., Jain, S., Song, H., Fu, M., Heuckeroth, R.O., Erlich, J.M., Jay, P.Y., and Milbrandt, J. (2007). Mice lacking sister chromatid cohesion protein PDS5B exhibit developmental abnormalities reminiscent of Cornelia de Lange syndrome. Development *134*, 3191-3201.

5. Maher, J.F., and Nathans, D. (1996). Multivalent DNA-binding properties of the HMG-1 proteins. Proc Natl Acad Sci U S A *93*, 6716-6720.

6. Metcalf, C.E., and Wassarman, D.A. (2006). DNA binding properties of TAF1 isoforms with two AT-hooks. J Biol Chem *281*, 30015-30023.

7. Nasmyth, K., and Haering, C.H. (2005). The structure and function of SMC and kleisin complexes. Annu Rev Biochem *74*, 595-648.

8. Glynn, E.F., Megee, P.C., Yu, H.G., Mistrot, C., Unal, E., Koshland, D.E., DeRisi, J.L., and Gerton, J.L. (2004). Genome-wide mapping of the cohesin complex in the yeast Saccharomyces cerevisiae. PLoS Biol *2*, E259.

9. Wendt, K.S., Yoshida, K., Itoh, T., Bando, M., Koch, B., Schirghuber, E., Tsutsumi, S., Nagae, G., Ishihara, K., Mishiro, T., et al. (2008). Cohesin mediates transcriptional insulation by CCCTC-binding factor. Nature *451*, 796-801.

10. Zhao, K., Kas, E., Gonzalez, E., and Laemmli, U.K. (1993). SAR-dependent mobilization of histone H1 by HMG-I/Y in vitro: HMG-I/Y is enriched in H1-depleted chromatin. Embo J *12*, 3237-3247.

11. Aulner, N., Monod, C., Mandicourt, G., Jullien, D., Cuvier, O., Sall, A., Janssen, S., Laemmli, U.K., and Kas, E. (2002). The AT-hook protein D1 is essential for Drosophila melanogaster development and is implicated in position-effect variegation. Mol Cell Biol *22*, 1218-1232.

12. Li, H., Coghlan, A., Ruan, J., Coin, L.J., Heriche, J.K., Osmotherly, L., Li, R., Liu, T., Zhang, Z., Bolund, L., et al. (2006). TreeFam: a curated database of phylogenetic trees of animal gene families. Nucleic Acids Res *34*, D572-580.

13. Panizza, S., Tanaka, T., Hochwagen, A., Eisenhaber, F., and Nasmyth, K. (2000). Pds5 cooperates with cohesin in maintaining sister chromatid cohesion. Curr Biol *10*, 1557-1564.

14. Andrade, M.A., Ponting, C.P., Gibson, T.J., and Bork, P. (2000). Homology-based method for identification of protein repeats using statistical significance estimates. J Mol Biol *298*, 521-537.

15. Carroll, S.L., Schweitzer, J.B., Miller, M.L., Sclar, G.M., and Milbrandt, J. (1993). Elements in the 5' Flanking Sequences of p75 NGF Receptor Gene Direct Appropriate CNS, But Not PNS, Expression in Transgenic Mice. Neuron.

16. Losada, A., Yokochi, T., and Hirano, T. (2005). Functional contribution of Pds5 to cohesin-mediated cohesion in human cells and Xenopus egg extracts. J Cell Sci *118*, 2133-2141.

Supplementary figure legends

Figure S1. Generation of *Pds5A*-deficient mice.

(**A**) Schematic representation of wildtype and genetrap alleles of *Pds5A* gene. The *pGT0lxf* gene trap vector is inserted in the second intron, which follows the first coding exon (encodes first 45 residues). Open boxes with numbers indicate exons (exon 1 is non-coding) and P1-P3 indicate positions of primers used for genotyping. A NEO probe was used for Southern analyses. SA, splice acceptor; S, stop codon; pA, polyadenylation site; pUC, vector backbone; MTF, marker fusion transcript. (**B**) Southern blot analysis using genomic DNAs from tails of wildtype (+/+) and *Pds5A* heterozygote (+/-) mice detected only one band for each restriction enzyme digest, indicating that only one copy of *pGT0lxf* is inserted in this mutant line. ND, no DNA loaded for that well. The DNA samples were digested with *Pst*I, *Bgl*II, *EcoR*V, *Nco*I, and *Ase*I, and hybridized with the radiolabeled DNA probe containing the NEO open reading frame indicated in A. (**C**) Sequencing trace showing the mapped *pGT0lxf* vector and genomic DNA junction using modified splinkerette PCR. (**D**) PCR genotyping analysis of the *Pds5B* mutation was performed with primers P1, P2, and P3 indicated in A. (**E**) Western blot of E18.5 kidney lysates probed with antibodies to PDS5A, β-GAL (β-galactosidase), and β-TUB (β-tubulin). Note the absence of PDS5A in *Pds5A*$^{-/-}$ lysates. No β-GAL fusion protein was present in wildtype lysates but was detected in lysates derived from *Pds5A* heterozygous and homozygous mice.

Figure S2. Expression of *Pds5A* and *Pds5B* in mouse embryonic tissues. Quantitative RT-PCR analysis of *Pds5B* and *Pds5A* mRNA levels in E14.5 mouse embryonic tissues normalized to *18S* RNA. Relative amounts of message RNA (mRNA) in different tissues were normalized to the mRNA level in liver.

Figure S3. Expression of *Pds5A* in adult mouse tissues. (**A**) Quantitative RT-PCR analysis of *Pds5A* mRNA levels in adult mouse tissues normalized to *18S* RNA. Error bars represent ± s.e.m. (**B-G**) β–galactosidase staining of tissues from adult *Pds5A*$^{+/-}$ mice. Note the high expression in Purkinje cells of the cerebellum (arrows in B), brain cortex (C), retina (D), periphery of seminiferous tubules (E), renal tubules (F), and medulla of thymus (dashed oval in G).

Figure S4. *Pds5A*-deficient mice have normal germ cell development. (**A-B**) H&E staining of germ cells of wildtype (**A**) and *Pds5A*$^{-/-}$ (**B**) neonatal ovaries show similar numbers of germ cells. Arrows point to germ cells with condensed chromosomes indicating meiotic prophase. (**C, D**) H&E staining of 4-week testes transplants of E18.5 wild-type (C) and *Pds5A*$^{-/-}$ mice (D). Note the presence of spermatogonia and spermatocytes in the periphery of wildtype and *Pds5A*-deficient seminiferous tubules. Round spermatids (arrows) are present in both wildtype and *Pds5A*$^{-/-}$ testis explants. (**E, F**) High magnification of 6-week testes transplants in Fig. 3G, H. Note the presence of Sertoli cells, spermatogonia, spermatocytes, round spermatids, and elongated spermatids in both wildtype and *Pds5A*-deficient testis transplants. StC, sertoli cells; SG, spermatogonia; SC, spermatocytes; RS, round spermatids; ES, elongated spermatids.

Figure S5. Normal chromatin-association dynamics of SCC1 in *Pds5A*$^{-/-}$*;Pds5B*$^{+/-}$ and *Pds5A*$^{+/-}$ *;Pds5B*$^{-/-}$ MEFs.

(**A**) FRAP analysis of SCC1-EGFP in wild type**,** *Pds5A*$^{-/-}$*;Pds5B*$^{+/-}$**,** and *Pds5A*$^{+/-}$*;Pds5B*$^{-/-}$ MEFs. MEF cells were infected with lentivirus expressing SCC1-EGFP. Half of the nucleus was bleached. The fluorescence intensity was measured every 30 sec for 30 min. The graphs show the decrease in the difference of fluorescence between the bleached and unbleached halves of the nuclei over time. Each curve is an average of at least eighteen cells. Note that fluorescence recovery dynamics of SCC1 is similar among all three MEF lines. (**B**), By fitting a bi-

exponential function to the fluorescence redistribution curve of SCC1-EGFP, we determined residence times of both weak and strong chromatin SCC1-EGFP. Note that there is no significant difference in the residence times of either weak or strong chromatin-bound SCC1-EGFP between wildtype and *Pds5* mutant MEFs. Residence times of strong chromatin-bound SCC1-EGFP are 1321 ± 116 sec (wild-type, n = 20), 1348 ± 113 sec ($Pds5A^{-/-}$;$Pds5B^{+/-}$, n = 18), 1133 ± 135 sec ($Pds5A^{+/-}$;$Pds5B^{-/-}$, n = 21) (mean ± SE). Residence times of weak chromatin-bound SCC1-EGFP are 32 ± 4 sec (wild-type, n = 20), 29 ± 3 sec ($Pds5A^{-/-}$;$Pds5B^{+/-}$, n = 18), 40 ± 6 sec ($Pds5A^{+/-}$;$Pds5B^{-/-}$, n = 21) (mean ± SE). (**C**) The soluble fraction of SCC1-EGFP was determined by the reduction of fluorescence after photobleaching. Fractions of weak and strong chromatin-bound components of SCC1-EGFP were determined by bi-exponential function fitting analysis of the redistribution curve. Note that there are no significant differences in the fractions of soluble, weak, and strong chromatin-bound SCC1-EGFP between Pds5 mutant and wildtype MEFS, although *Pds5* mutant MEFs do show a trend toward increased strong chromatin-bound SCC1-EGFP as demonstrated previously [16].

Figure S6. Evolution of PDS5 and identification of AT hook domain in PDS5A.

(**A**). Phylogenetic tree of PDS5. One gene encoding a PDS5 protein is present from yeast to fly, whereas two PDS5 homologs are present in vertebrate genomes. Scale bar = 0.1 amino acid substitutions per site. ARATH, *Arabidopsis thaliana*; SCHPO, *Schizosaccharomyces pombe*; HUMAN, *Homo sapiens;* PANTR, *Pan troglodytes;* CANFA, *Canis familiaris;* MOUSE, *Mus musculus;* RAT, *Rattus norvegicus;* CHICK, *Gallus gallus;* XENTR, *Xenopus tropicalis;* BRARE, *Danio rerio;* TETNG, *Tetraodon nigroviridis*; FUGRU, *Fugu rubripes*; APIME, *Apis mellifera*; DROME, *Drosophila melanogaster*; ANOGA, *Anopheles gambiae*; CAEBR, *Caenorhabditis briggsae*; CAEEL, *Caenorhabditis elegans*. (**B**). Schematic protein domain

structure of human PDS5A and PDS5B. Red rectangle represents one HEAT repeat. Gray rectangle is a leucine zipper domain. Blue rectangle represents an AT-hook domain. (**C**) Multispecies alignment of AT-hook domain identified in PDS5A and PDS5B. *Hs, Homo sapiens; Pa, Pan troglodytes; Ca, Canis familiaris; Mm, Mus musculus; Rn, Rattus norvegicus; Ch, Gallus gallus; Xe, Xenopus tropicalis; Br, Danio rerio; Te, Tetraodon nigroviridis; Fu, Fugu rubripes*. Note that AT-hook domains from all species listed here have an invariant core sequence (RGRP), except for mammalian PDS5A where the Pro has been substituted by Arg at the 4th position.

Figure S7. SNPs detected in human PDS5B gene.

The diagram of PDS5B gene structure with 36 exons (red, coding region; blue, untranslated region) (http://www.ncbi.nlm.nih.gov; GeneID: 23047). The positions of common SNPs are annotated with vertical black bars with numbers. The table below lists the SNP name, type, ID (if it has been reported before), and frequency observed in this study.

Figure S8. SNPs detected in human PDS5A gene.

The diagram of PDS5A gene structure with 33 exons (red, coding region; blue, untranslated region) (http://www.ncbi.nlm.nih.gov; GeneID: 23244). The positions of SNPs are annotated with vertical black bars with numbers. The table below lists the SNP name, type, ID (if it has been reported before), and frequency observed in this study

Figure S9. The proband with the PDS5B(R1292Q) mutation shows dysmorphic facial features consistent with CdLS. The proband (individual II-2 in figure 7D) shows microbrachycephaly, arched eye brows, long eyelashes, synophrys, ptosis, wide-spaced teeth, a thin down-turned upper lip and a long philtrum.

Figure S10. Purification of PDS5B proteins and the sequence of AT-rich DNA bound by PDS5B. (**A**) Diagram of His-tagged carboxy terminus of PDS5B containing two AT hook domains (C-PDS5B-His), which was expressed in *E.coli* and purified by Ni-affinity chromatography. (**B**) Commassie Blue staining of purified C-PDS5B-His proteins for relative protein quantification. (**C**) Sequences of DNA probe used for EMSA. (**D**) EMSA demonstrating specific DNA binding of PDS5B. ^{32}P-labeled T5-8-T5 probe (in C) forms complexes with *wild type* PDS5B protein and the interaction is competed by specific competitors (SC) but not by non-specific competitors (NSC). SC, cold T5-8-T5 probe; NSC, cold T5-8-T5 mutant probe (T5-8-T5 mutant forward:5'-GGACTCCAGGTCCAGGACCGCTcgcgGCGCGCGCcgcgTGCGGGAGGTCCAGCTGTCCACCTCC-3' and 5'-GGAGGTGGACAGCTGGACCTCCCGCAcgcgGCGCGCGCcgcgAGCGGTCCTGGACCTGGAGTCC-3'). Arrows, protein-DNA complexes.

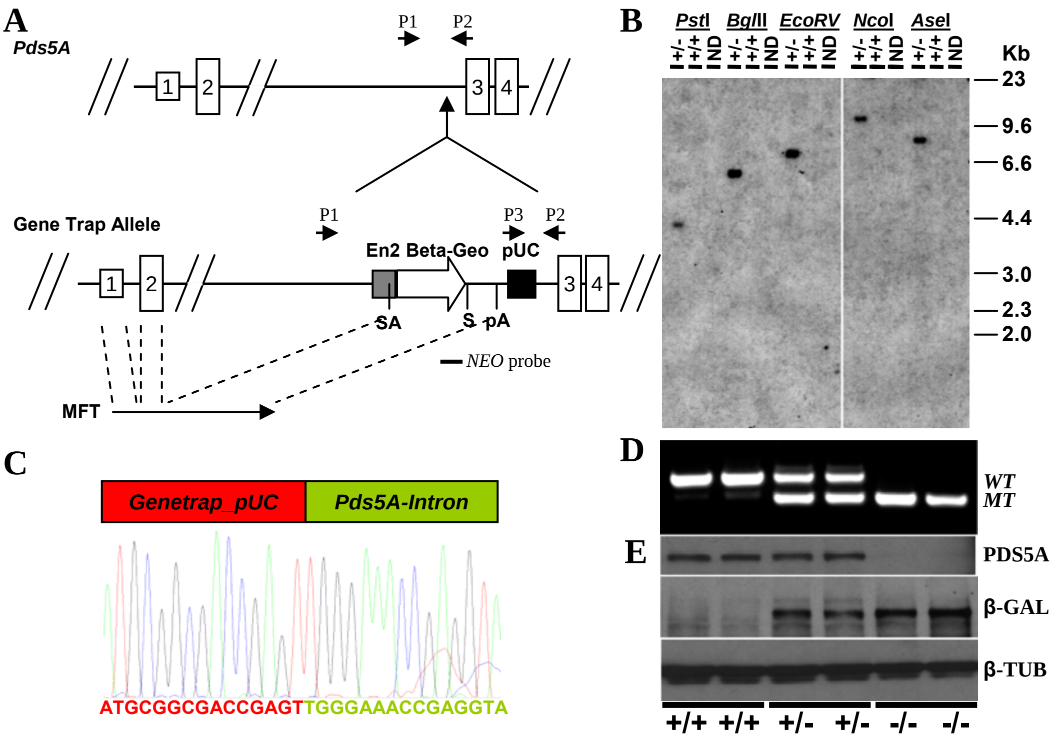

Figure S1
Zhang B et al.

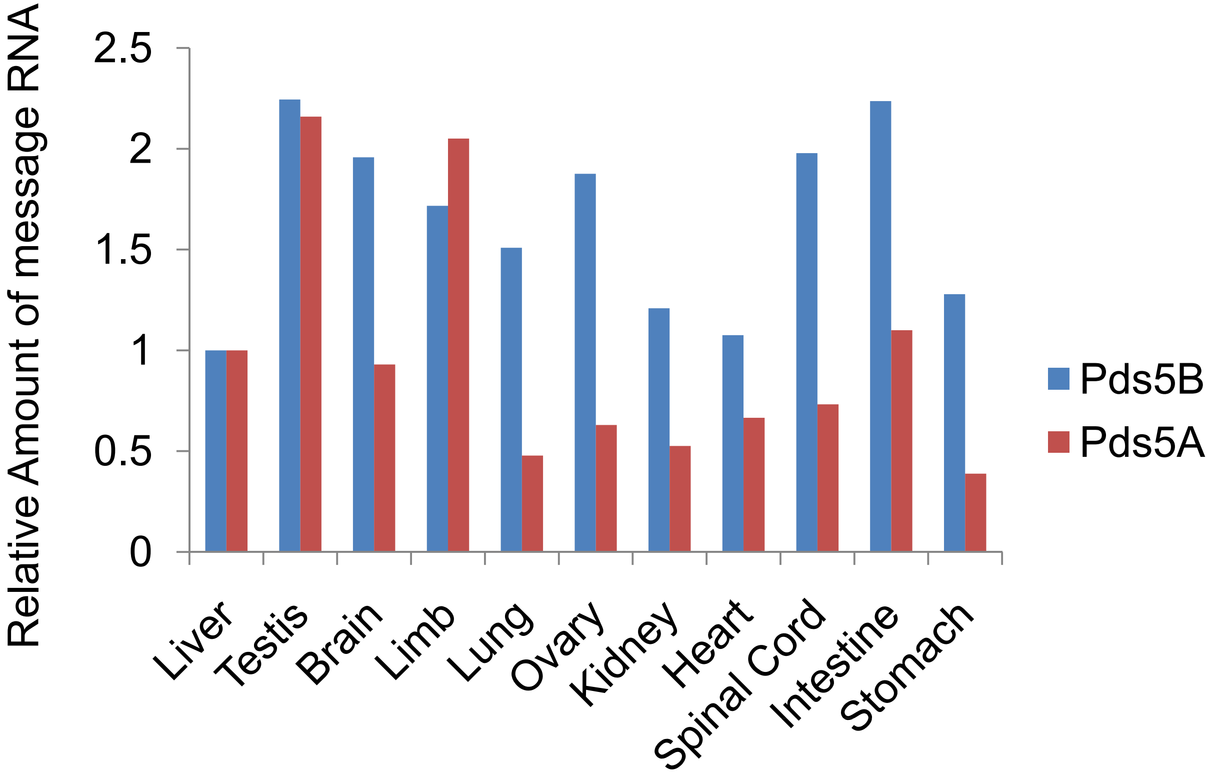

Figure S2
Zhang B et al.

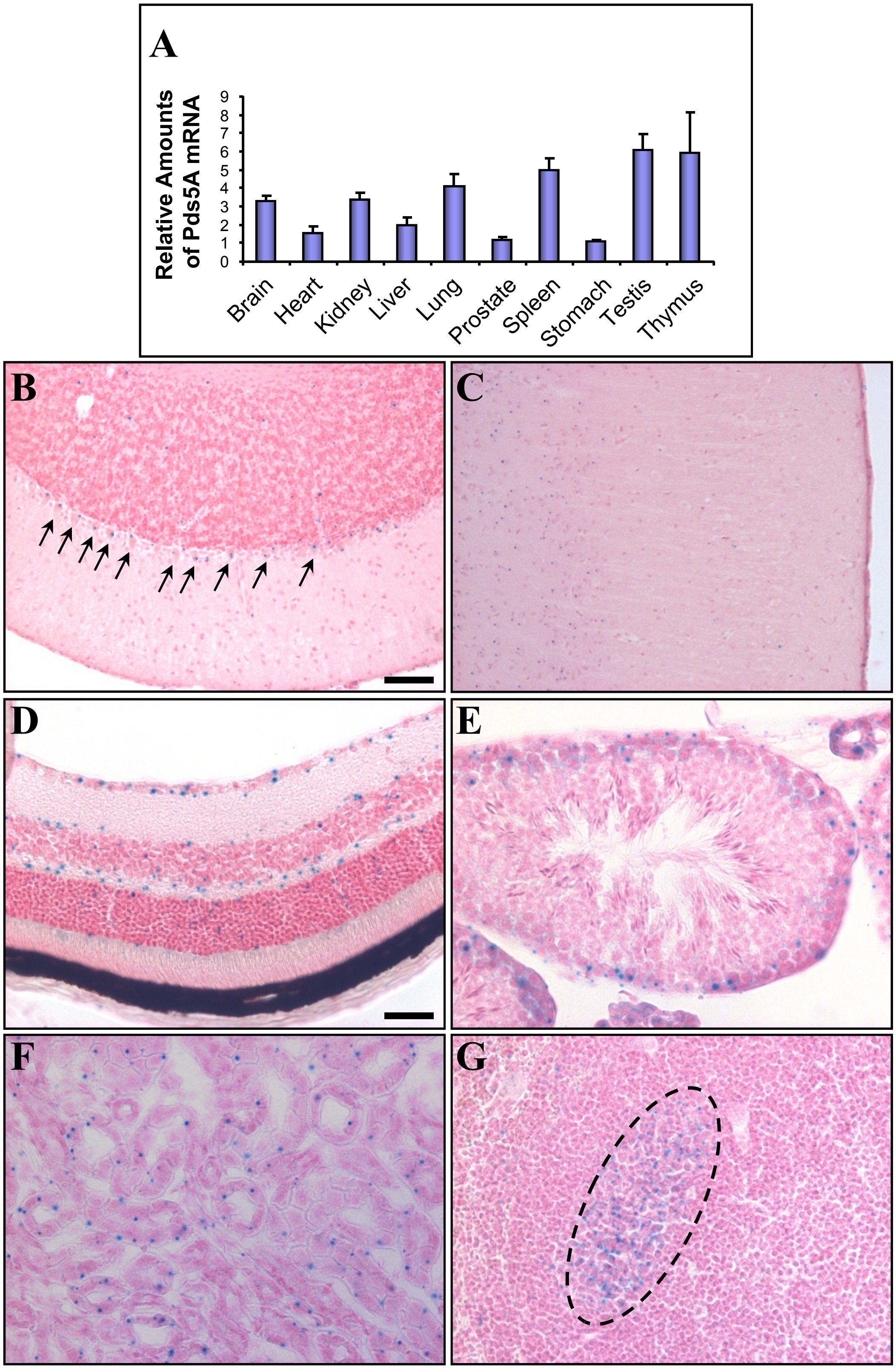

Figure S3
Zhang B et al.

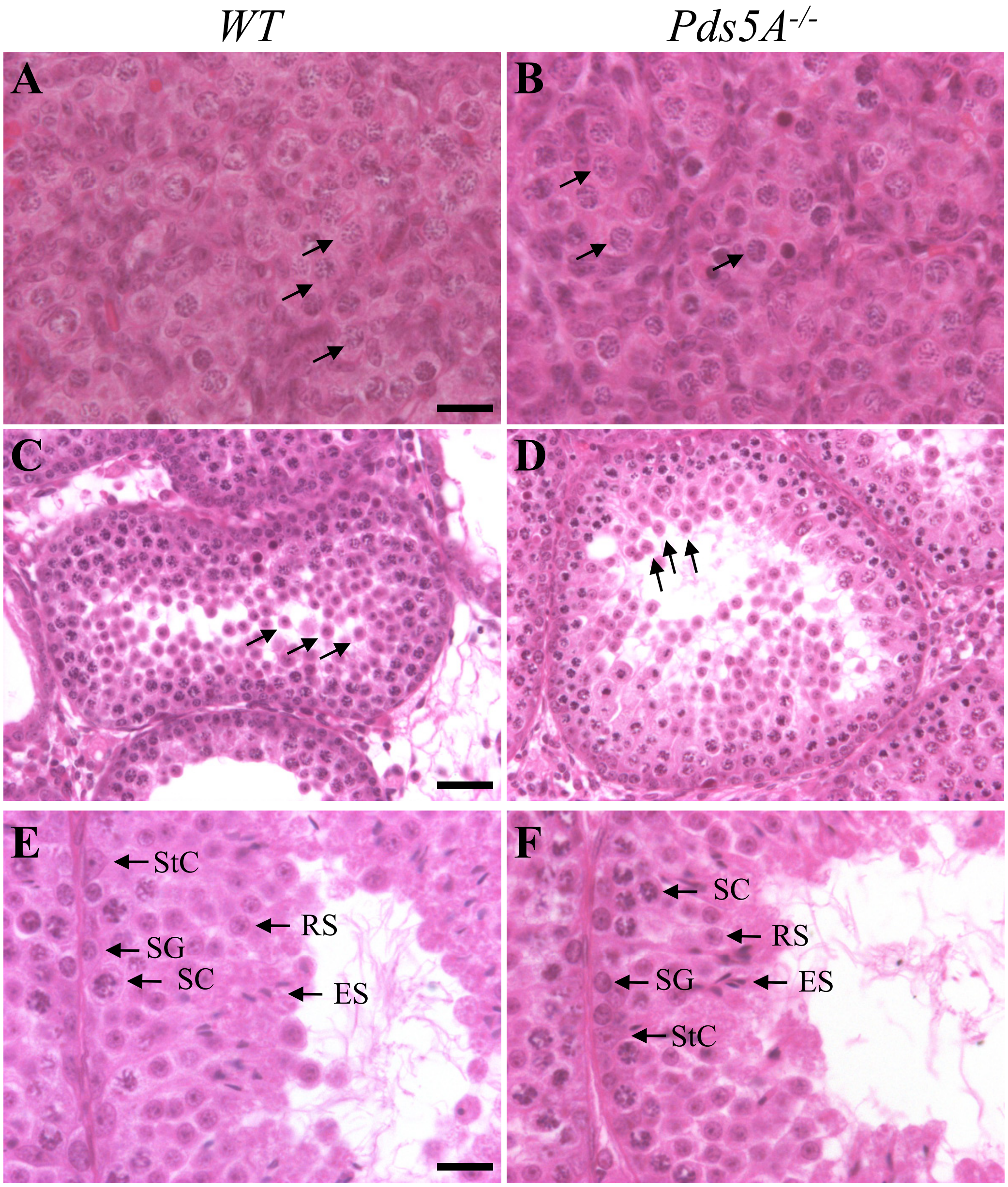

Figure S4
Zhang B et al.

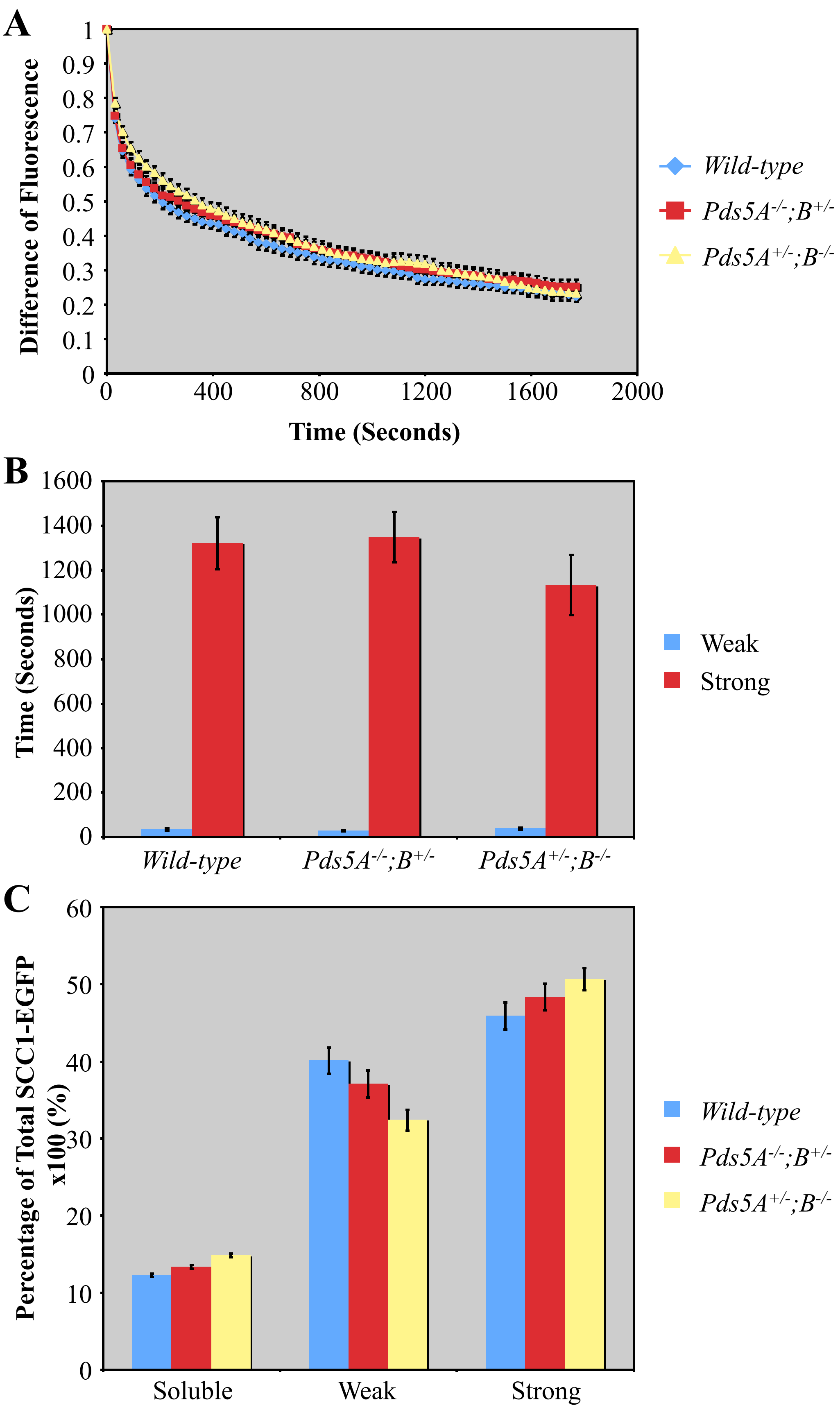

Figure S5
Zhang B et al.

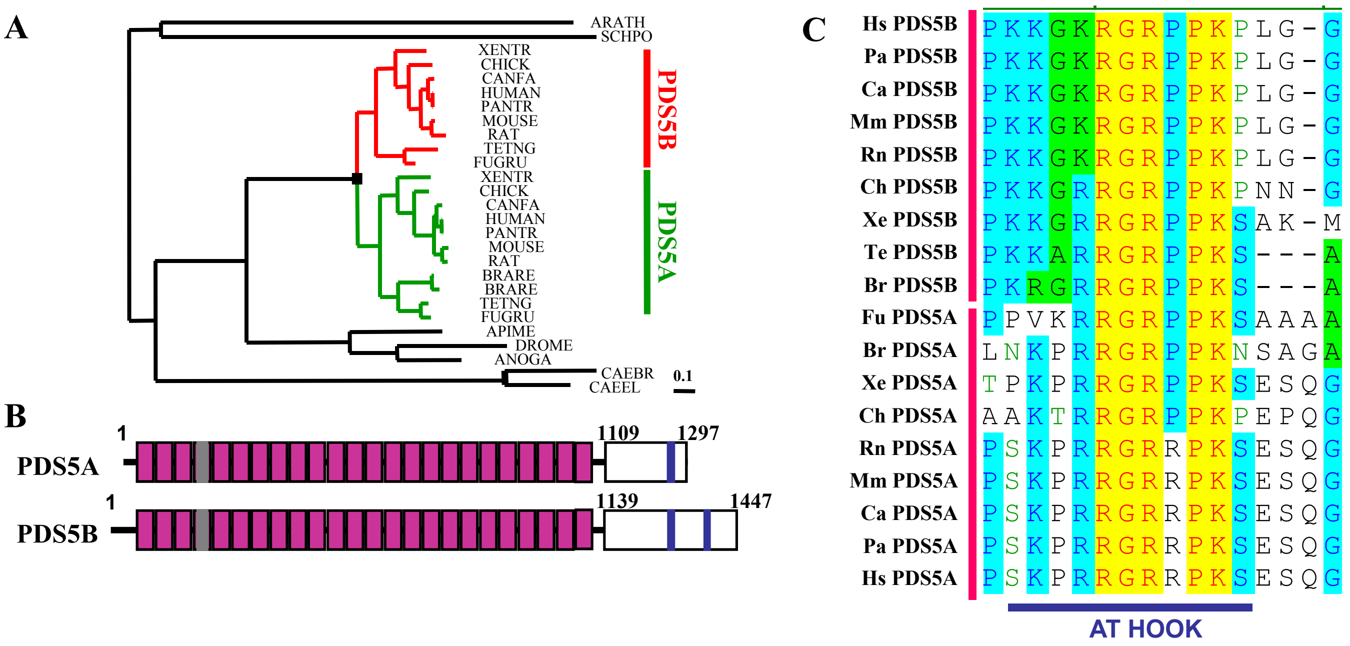

Figure S6
Zhang B et al.

Figure S7
Zhang B et al.

PDS5B: 23047 (Gene ID)

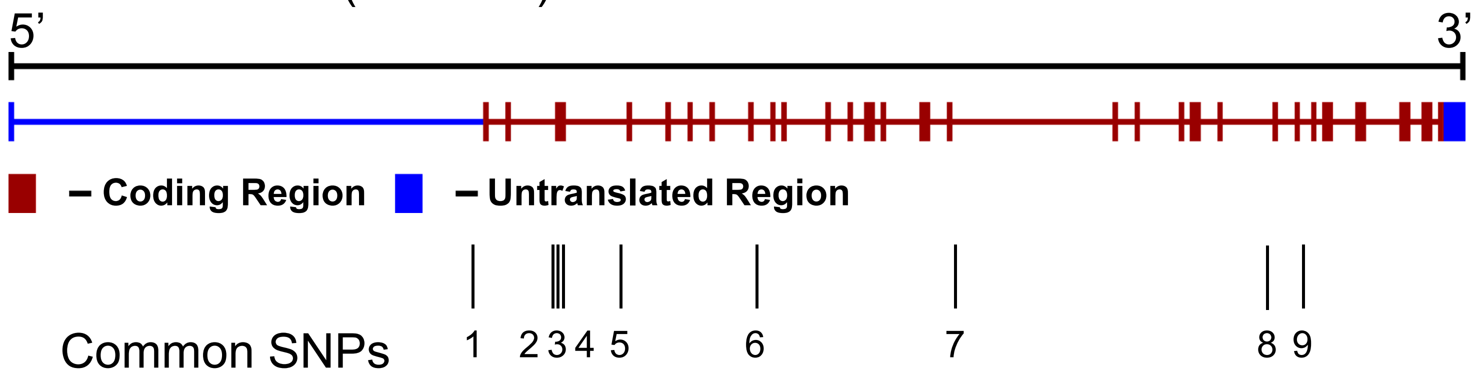

SNP number	SNP name	SNP type	SNP ID	SNP frequency
1	**-79g>a**	intronic	rs2301389	A: 0.486; G: 0.514
2	**313-42g>a**	intronic	rs7335546	A: 0.486; G: 0.514
3	**372a>g**	nonsynonymous	rs2301393	A: 0.519; G: 0.481
4	**400-47t>c**	intronic	Not reported	T: 0.977; C: 0.023
5	**498-132a>g**	intronic	Not reported	A: 0.771; G: 0.229
6	**1057+78a>-**	intronic	rs11355777	A: 0.529; -: 0.471
7	**2407-12g>a**	intronic	rs733487	A: 0.515; G: 0.485
8	**3190-24g>t**	intronic	rs3752474	G: 0.519; T: 0.481
9	**3372+48a>c**	intronic	rs7988089	A: 0.514; C: 0.486
10	**-23t>a**	intronic	Not reported	Once
11	**2736c>t**	nonsynonymous	Not reported	Once
12	**3309+15a>g**	intronic	Not reported	Once
13	**4257c>t**	nonsynonymous	Not reported	Once

Figure S8
Zhang B et al.

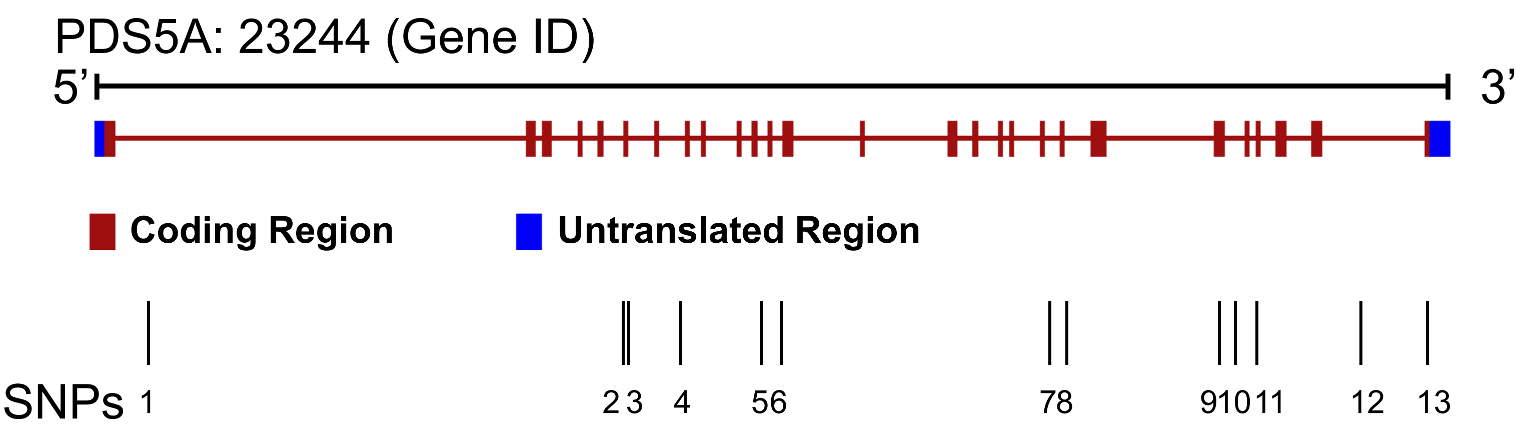

SNP number	SNP name	SNP type	SNP ID	SNP frequency
1	**18+55t>C**	intronic	rs35426579	T: 0.785; C: 0.215
2	**535-23t>G**	intronic	rs28419175	T: 0.939; G: 0.061
3	**612t>C**	nonsynonymous	rs28449663	T: 0.627; G: 0.373
4	**757-108c>G**	intronic	rs11725674	C: 0.728; G: 0.272
5	**1266-24t>A**	intronic	Not reported	T: 0.987; A: 0.013
6	**1379+43c>T**	intronic	Not reported	C: 0.921; T: 0.079
7	**2157+35c>T**	intronic	rs28546862	C: 0.921; T: 0.079
8	**2652g>A**	nonsynonymous	Not reported	Once
9	**3112g>A (V1038I)**	synonymous	Not reported	Once
10	**3219+27a>G**	intronic	Not reported	A: 0.987; G: 0.013
11	**3283-54a>G**	intronic	Not reported	Once
12	**3891-20g>a**	intronic	Not reported	Once
13	***204g>c**	3' UTR	Not reported	Once

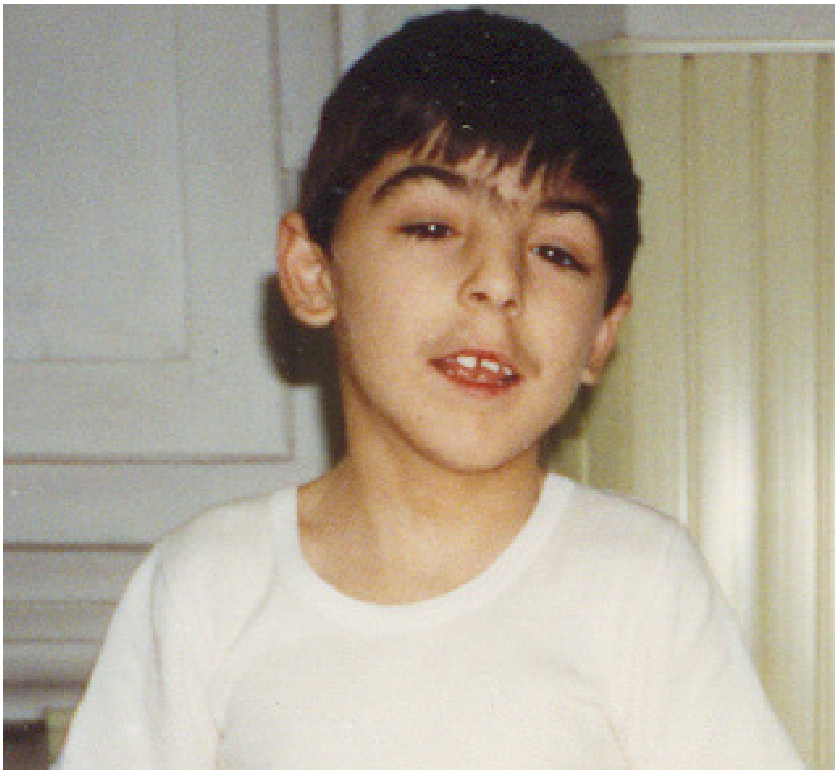

Figure S9
Zhang B et al.

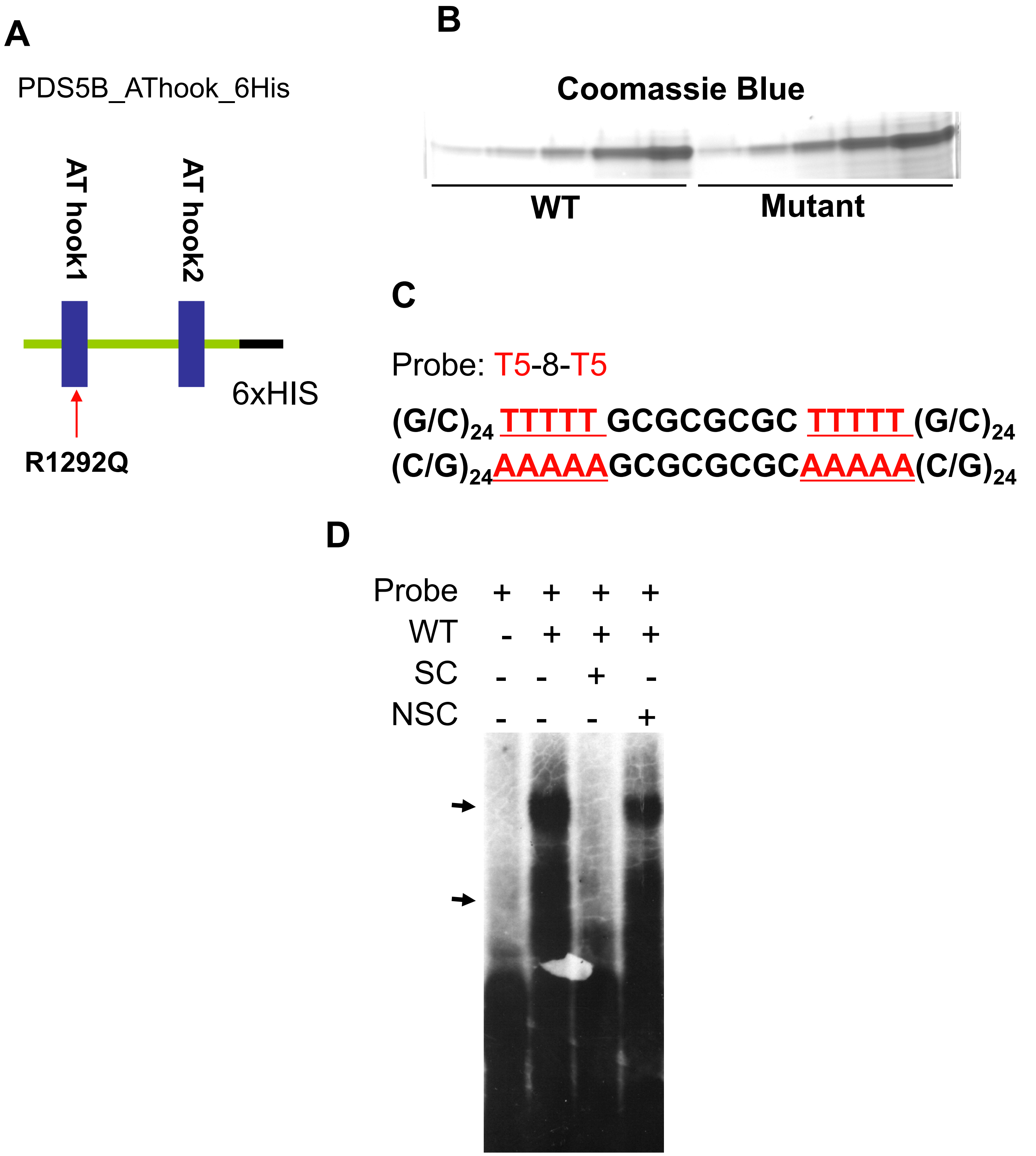

Figure S10
Zhang B et al.

Table S1: Summary of congenital heart defects in *Pds5A*$^{-/-}$ mice.

	ASD	TOF	VSD	AVCD	DORV	Total
Wild-type	**0/18**	**0/18**	**3/18**	**0/18**	**0/18**	**3/18**
Pds5A $^{+/-}$	**3/19**	**1/19**	**1/19**	**0/19**	**0/19**	**5/19**
Pds5A $^{-/-}$	**1/25**	**0/25**	**10/25**	**5/25**	**1/25**	**17/25**

ASD: Atrial septal defects
TOF: Tetrology of Fallot
VSD: Ventricular septal defects
AVCD: Atrioventricular canal defects
DORV: Double outlet right ventricle
$P<0.001$ is calculated by a Z-test for significant differences in total incidence between *WT* and *Pds5A* homozygotes.

Table S2: Primers used for resequencing human PDS5B gene.

Number	Gene Name	Entrez Gene ID	Forward Primer	Reverse Primer
1	PDS5B	23047	tgtaaaacgacggccagtggttggtggggaaggttacc	caggaaacagctatgacccttagatcttttctatgccatttgctg
2	PDS5B	23047	tgtaaaacgacggccagtgaagttaaccttagcaattaacatgacc	caggaaacagctatgaccgtcaaagaatccaaggaaatcatct
3	PDS5B	23047	tgtaaaacgacggccagtcatgagtgctgaataaactcttgg	caggaaacagctatgacctgttatatgacttgacccaagcaa
4	PDS5B	23047	tgtaaaacgacggccagtgctagaggatacaaagagcccac	caggaaacagctatgaccctcagccttctaatattcattgtaacag
5	PDS5B	23047	tgtaaaacgacggccagttatgatgtgggagagagggaagc	caggaaacagctatgaccgcaggataaaccatatttagaacctaag
6	PDS5B	23047	tgtaaaacgacggccagtgtggttttgccttctctcttttattc	caggaaacagctatgaccggatgagagcaagccttcca
7	PDS5B	23047	tgtaaaacgacggccagtgtctgttttattgtatacaagatggattcc	caggaaacagctatgacccacaaacagacacggcaca
8	PDS5B	23047	tgtaaaacgacggccagtctgtggaattggagcatcagg	caggaaacagctatgaccagccagtcattaacaactttcaaaact
9	PDS5B	23047	tgtaaaacgacggccagtgtttggttctactttgcattttcct	caggaaacagctatgaccatgaccttaaactaaaacaagacttcacc
10	PDS5B	23047	tgtaaaacgacggccagtctatgtaggtattgttttgctagggct	caggaaacagctatgacccctccatacccctaaaaaaggc
11	PDS5B	23047	tgtaaaacgacggccagtgggaaggaaaacacaaaactagga	caggaaacagctatgaccgagcccagtcactcactaccat
12	PDS5B	23047	tgtaaaacgacggccagtgagcggcaggaacttctttaata	caggaaacagctatgaccgccaagtactacgacataaccctttta
13	PDS5B	23047	tgtaaaacgacggccagttgcttagtcctgtgaatttcatctc	caggaaacagctatgacccaaggaatcctaacttctacaatcaga
14	PDS5B	23047	tgtaaaacgacggccagtagagcatgggtctgcagcattta	caggaaacagctatgacctttatgtactaaatcctttctccagctc
15	PDS5B	23047	tgtaaaacgacggccagtctgaatttgtacgtaaggattttaaggc	caggaaacagctatgaccgaaggacatacaccacaatattaacaattg
16	PDS5B	23047	tgtaaaacgacggccagtccagcgtcagtactacatggtttta	caggaaacagctatgacccacacagatgtgtagctagatatagcagg
17	PDS5B	23047	tgtaaaacgacggccagtgctatatcaaatttgtatttggttcgga	caggaaacagctatgacccaacttctaaatcatctaaaacaactgaga
18	PDS5B	23047	tgtaaaacgacggccagtcaatcttcctacagaggaacatttctg	caggaaacagctatgaccctcatgcatcttctaaaataccaatca
19	PDS5B	23047	tgtaaaacgacggccagtcataggcctttggaagtgagtttg	caggaaacagctatgaccgatgcatcaccaaatgtgaacat
20	PDS5B	23047	tgtaaaacgacggccagtgaacctgatgccttctggaca	caggaaacagctatgacccaaggcagtcaaatggaagtattattg
21	PDS5B	23047	tgtaaaacgacggccagttcactgcagaaggtacagatttcat	caggaaacagctatgacccattattaccctatagtctcacagcaca
22	PDS5B	23047	tgtaaaacgacggccagtcagcaaagaacaaactagtctttagtca	caggaaacagctatgaccagacatgtgaccgccactgta
23	PDS5B	23047	tgtaaaacgacggccagtgtgccagaatttttaataaaattagatgtacag	caggaaacagctatgaccctcaaatgacagtttcagaactctga
24	PDS5B	23047	tgtaaaacgacggccagtcactcttgtgagcagtgaagca	caggaaacagctatgacccagatataggttgatatttaagtaccatgcat
25	PDS5B	23047	tgtaaaacgacggccagtacgaaagaaagagtatgtaagatttggt	caggaaacagctatgaccgaacaccagactggtacagaaacataac
26	PDS5B	23047	tgtaaaacgacggccagtgtatgacatattctttttccctgctg	caggaaacagctatgaccgcaacagtctgtgtaagcatcttagc
27	PDS5B	23047	tgtaaaacgacggccagttgctattcagactggattcatgtt	caggaaacagctatgaccatgtgtgtcctccagtttgactc
28	PDS5B	23047	tgtaaaacgacggccagtctgctgtttgctttgttggct	caggaaacagctatgacccaggtctcctaggactttattacaagcta
29	PDS5B	23047	tgtaaaacgacggccagtctttgagagagatgttatttatggttctt	caggaaacagctatgaccccatccaaaatgagcaagca
30	PDS5B	23047	tgtaaaacgacggccagttccataaatctgtaaagctacactagga	caggaaacagctatgaccgaggataattataaactcaggtttgtgg

31	PDS5B	23047	tgtaaaacgacggccagtggtacatatctaggacaacattagataacag	caggaaacagctatgaccgaaacagatgcagtttagagttacacatg
32	PDS5B	23047	tgtaaaacgacggccagtctggacctccagcaccaga	caggaaacagctatgacccttacctagtgtaatggggctaaatatatgg
33	PDS5B	23047	tgtaaaacgacggccagtgggtaaagatgatatgacgatgaaa	caggaaacagctatgaccatgttctccattatccaacctgg
34	PDS5B	23047	tgtaaaacgacggccagttttcagggaaaggaaagcaaaga	caggaaacagctatgacctggctaaagcatgtgaccacaaa

Table S3: Primers used for resequencing human PDS5A gene.

Number	Gene Name	Entrez Gene ID	Forward Primer	Reverse Primer
1	PDS5A	23244	tgtaaaacgacggccagtcctgtgtaacatgggaattcctg	caggaaacagctatgacccaactatggaaatgaaatcattttcg
2	PDS5A	23244	tgtaaaacgacggccagtggtttctctctcaaatgcttaggtg	caggaaacagctatgaccagttaactggaagccctcaaatc
3	PDS5A	23244	tgtaaaacgacggccagtcatgtgcattgaagtgttggctg	caggaaacagctatgacccgaatcttgctattgttgcttca
4	PDS5A	23244	tgtaaaacgacggccagtgtaccagaacaccgctaatgaca	caggaaacagctatgacctgaacatttctggtaaatttaactaattagc
5	PDS5A	23244	tgtaaaacgacggccagtggctgctttagatgctttaaatcc	caggaaacagctatgaccgaataaactgcattgaaccgtatga
6	PDS5A	23244	tgtaaaacgacggccagtttactctgtggtcaagacaattcaa	caggaaacagctatgaccggctgcataaacagtgctgaaca
7	PDS5A	23244	tgtaaaacgacggccagtccaagtgggacaatatatttttctattaca	caggaaacagctatgacccagacaacagaaattcccagctaac
8	PDS5A	23244	tgtaaaacgacggccagtgaacaaccaaaacctggtagatgg	caggaaacagctatgaccgtatccaggtatcatgttgtgcct
9	PDS5A	23244	tgtaaaacgacggccagtgtacatcataactacaatgacagcattct	caggaaacagctatgaccctatggatacaaatccatttataatgtgaa
10	PDS5A	23244	tgtaaaacgacggccagtggtaacactcatcctacagcagca	caggaaacagctatgacctgtgttaagttcatggactcaaagtagc
11	PDS5A	23244	tgtaaaacgacggccagtgagtacataagatctgaccctgaagatc	caggaaacagctatgaccgtctgctgatggagatgagtaatctc
12	PDS5A	23244	tgtaaaacgacggccagtggtgaggaagataactacaaaatgtcc	caggaaacagctatgacccaagctctggttggtgatgtt
13	PDS5A	23244	tgtaaaacgacggccagtgagaattacacagaaacagaagtgtttct	caggaaacagctatgaccgaagattggtttgggcatggt
14	PDS5A	23244	tgtaaaacgacggccagtaggacttcatataaacagctttaaaaaatct	caggaaacagctatgaccgccttctctactagcatccatcc
15	PDS5A	23244	tgtaaaacgacggccagtagcttctaaatcaaagttgtctttcttcc	caggaaacagctatgaccgcgggaagtaattcttttttaatgga
16	PDS5A	23244	tgtaaaacgacggccagtcaaatagtttcctgtgaaataagcctga	caggaaacagctatgaccgaagtttagttatcaagtagaggacaaggt
17	PDS5A	23244	tgtaaaacgacggccagttgaatgatgtacaaccctgtgatt	caggaaacagctatgacctctagagcagacaattcttaaccactt
18	PDS5A	23244	tgtaaaacgacggccagtgctgaataaatgaatgcataagttttcc	caggaaacagctatgaccgagaatttgcctgaccccg
19	PDS5A	23244	tgtaaaacgacggccagtgcttgtttgcaagaacaggttg	caggaaacagctatgaccattttccattgagcagactgtgat
20	PDS5A	23244	tgtaaaacgacggccagtggaaatttagcttggacaactacaga	caggaaacagctatgaccccccggtatttaaacttttaggtagatgtag
21	PDS5A	23244	tgtaaaacgacggccagtgacctggctgccaaacaag	caggaaacagctatgaccgcttcttaaaaatggaataattgtgtca
22	PDS5A	23244	tgtaaaacgacggccagtcgtggacagtcttgtttaaacaca	caggaaacagctatgaccgtgtgccagtttataaaggcaca
23	PDS5A	23244	tgtaaaacgacggccagtgcaggaaacatacaaattaggagctc	caggaaacagctatgacctgtgattacagtagagtactgctgctgta
24	PDS5A	23244	tgtaaaacgacggccagtaacacgagaccataccagagttaaa	caggaaacagctatgacccccttcagaataccttagctcataacaa
25	PDS5A	23244	tgtaaaacgacggccagtgaaggatggaaacacagatgaca	caggaaacagctatgacccaatttctgcatagtaagtgaggacatatg

26	PDS5A	23244	tgtaaaacgacggccagtttcctggaaagtttaaagggcat	caggaaacagctatgacccctgtttgtatcacctccttcaat
27	PDS5A	23244	tgtaaaacgacggccagtcgtcatgaaacttatgctgcatt	caggaaacagctatgaccgcttcatattttagtgaagcaaaattca
28	PDS5A	23244	tgtaaaacgacggccagtaattacatgggattgcaaatggc	caggaaacagctatgaccttattggcactgtccctcaagtg
29	PDS5A	23244	tgtaaaacgacggccagtactttcccctttatgttaagttttctaagg	caggaaacagctatgacccagtttgagaaaattgatgccatc
30	PDS5A	23244	tgtaaaacgacggccagtccatcagaatatactttatctcttcaggatag	caggaaacagctatgaccgattaggccaatcccaagaca
31	PDS5A	23244	tgtaaaacgacggccagtcaaacaaccaagcaacccag	caggaaacagctatgaccgttgactgtgaactgtctggtggc
32	PDS5A	23244	tgtaaaacgacggccagtgagatacaacagtacctttcagaggg	caggaaacagctatgacctagaggccggtagaggactgtgaac

Table S4: SNPs analysis for PDS5B gene in members of the familial case of CdLS.

			**Patient**	**I-1: CDL-238F**			**I-2: CDL-238M**			**II-2:CDL-238P**			**II-3: CDL-238AS**			**II-4: CDL-238B**		
				**Allele**														
**Gene**	**SNPs**	**Chrom**	**Position**	g	p1	p2	g	m1	m2	g	m	p	g	m	p	g	m	p
PDS5B	rs7328733	13	32024737	BB	B	B	AA	A	A	AB	A	B	AB	A	B	AB	A	B
PDS5B	rs208428	13	32030911	AA	A	A	AB	B	A	AB	B	A	AB	B	A	AA	A	A
PDS5B	rs7332115	13	32045548	BB	B	B	AA	A	A	AB	A	B	AB	A	B	AB	A	B
PDS5B	rs208418	13	32052022	AA	A	A	AB	A	B	AA	A	A	AA	A	A	AB	B	A
PDS5B	rs9535012	13	32068934	AA	A	A	AB	B	A	AB	B	A	AB	B	A	AA	A	A
PDS5B	rs9595908	13	32082288	BB	B	B	AA	A	A	AB	A	B	AB	A	B	AB	A	B
PDS5B	rs208426	13	32089371	BB	B	B	AB	B	A	BB	B	B	BB	B	B	AB	A	B
PDS5B	rs2301390	13	32121444	BB	B	B	AA	A	A	AB	A	B	AB	A	B	AB	A	B
PDS5B	rs12428283	13	32125912	BB	B	B	AA	A	A	AB	A	B	AB	A	B	AB	A	B
PDS5B	rs590383	13	32161714	AA	A	A	AB	A	B	AA	A	A	AA	A	A	AB	B	A
PDS5B	rs4120131	13	32175086	AA	A	A	BB	B	B	AB	B	A	AB	B	A	AB	B	A
PDS5B	rs658996	13	32178675	BB	B	B	AB	A	B	AB	A	B	AB	A	B	BB	B	B
PDS5B	rs506645	13	32205709	AA	A	A	AB	A	B	AA	A	A	AA	A	A	AB	B	A
PDS5B	rs7985485	13	32258474	BB	B	B	AB	A	B	AB	A	B	AB	A	B	BB	B	B
PDS5B	rs17077875	13	32274118	AA	A	A	AA	A	A	AA	A	A	AA	A	A	AA	A	A
PDS5B	rs4942925	13	32279244	BB	B	B	AA	A	A	AB	A	B	AB	A	B	AB	A	B
PDS5B	rs7331256	13	32281103	BB	B	B	BB	B	B	BB	B	B	BB	B	B	BB	B	B
PDS5B	rs17077900	13	32283927	AA	A	A	AA	A	A	AA	A	A	AA	A	A	AA	A	A
PDS5B	rs990324	13	32285246	AB	A	B	AA	A	A	AB	A	B	AB	A	B	AB	A	B
PDS5B	rs2320611	13	32293373	BB	B	B	BB	B	B	BB	B	B	BB	B	B	BB	B	B

g, genotype; p1,p2, paternal alleles; m1, m2, maternal alleles ; m, maternal allele; p, paternal allele.
Note that a maternal *PDS5B* m1 allele (pink) is shared among the two affected siblings (II-2 and II-3) and not by the unaffected brother (II-4). The paternal *PDS5B* alleles are distinguished only by a single SNP, rs990324, and presumably the same p2 paternal allele (lighter blue). is shared among all 3 children
